# Anisotropic N-Graphene-diffused Co_3_O_4_ nanocrystals with dense upper-zone top-on-plane exposure facets as effective ORR electrocatalysts

**DOI:** 10.1038/s41598-018-21878-w

**Published:** 2018-02-27

**Authors:** D. Hassen, M. A. Shenashen, A. R. El-Safty, A. Elmarakbi, S. A. El-Safty

**Affiliations:** 1 National Institute for Materials Science (NIMS), Research Center for Strategic Materials, 1-2-1 Sengen, Tsukuba-shi, Ibaraki-ken 305-0047 Japan; 2 Faculty of Engineering and Advanced and Manufacturing, University of Sunderland, St Peter’s Campus, Sunderland, SR6 0DD UK

## Abstract

We provide strong evidence of the effectiveness of homogenously self-propelled particle-in-particle diffusion, interaction and growth protocol. This technique was used for one-pot synthesis of novel nitrogen-graphene oxide (N-GO)/Co_3_O_4_ nanocrystals with cuboid rectangular prism-shaped nanorods (NRs) along {110}-plane and truncated polyhedrons with densely-exposed, multi-facet sites along {311} and {111} planes. These hierarchal nanocrystals create electrode catalyst patterns with vast-range accessibility to active Co^2+^ sites, a vascular system for the transport and retention of captured O_2_ molecule interiorly, and low adsorption energy and dense electron configuration surfaces during the oxygen reduction reaction (ORR). The superior electrocatalytic ORR activity of the N-GO/Co_3_O_4_ polyhedron nanocrystals in terms of electrochemical selectivity, durability and stability compared with NRs or commercial Pt/C catalysts confirms the synergetic contribution of multi-functional, dense-exposed, and actively topographic facets of polyhedrons to significantly activate the catalytic nature of the catalyst. Our findings show real evidence, for the first time that not only the large number of catalytically active Co^2+^ cations at the top surface layer but also the dense location of active Co^2+^ sites on the upper-zone top-on-plane exposure, and the electron density configuration and distribution around the Co^2+^ sites were important for effective ORR.

## Introduction

Oxygen reduction reaction (ORR) is an important cathodic reaction in energy conversion and storage technologies, particularly in low-temperature proton exchange membrane fuel cells (PEMFCs)^1–4^. The high-efficiency PEMFCs synthesis/design is dependent on the electroactive materials used in the ORR process^5^. Platinum-based electrocatalysts have been extensively investigated as ORR catalysts because of their excellent electrocatalytic performance^6,7^. However, there are various fundamental obstacles which curbed the wide-scale fuel cells commercialization using Pt-catalysts such as the poor stability, high cost, and sluggish kinetics^8,9^. Therefore, furthering fuel cell research requires the development of cost-effective and efficient electrocatalysts for ORR based on non-precious metals^10^. Earth-abundant transition metals and their alloys are considered highly promising electroactive materials that exhibit enhanced ORR activities^11–13^. Among these materials, cobalt-based oxides have recently received particular interest because of their potentially improved electrocatalytic performance, unique redox features, and easy preparation^14–17^.

Supports such as electron-rich nitrogen materials into the hierarchal structure of Co -based catalysts have been proven advantageous, as they transform Co -based catalysts into excellent ORR catalysts^17^. Graphene sheets are regarded as a promising support over the rest because of their extensive applicability in green energy technologies^18,19^.

In addition to hydrothermal growth, state-of-the-art design strategies have been investigated to improve the geometric and electronic features of metal mesocrystals and their derivatives and thus enhance their electrocatalytic performance toward ORR. The controlled design of shape-dependent heterogeneous catalysts with more reactive crystal facets and preferential high-surface-energy planes plays a crucial role in the commercial success of non-precious metal electrocatalysts^20,21^. Recently, experimental outcomes and theoretical analyses suggested that exposed energy facets are the main determining factors affecting the catalytic activity of these nanostructures^22,23^. Specifically, catalytic materials with similar chemical composition but with different well-defined planes exhibit distinct catalytic activities because of their varying atomic configuration and electronic structures^22,23^. Guo *et al*. reported the controllable synthesis of Co_3_O_4_ nanostructures with different reactive crystal planes, namely, {111}, {100}, and {110}, for ORR; results showed that the catalytic activity of the as-obtained architectures was surface dependent^24^. The exposed {111} surfaces were the most active sites due to abundant Co^2+^ sites^20^. Sun *et al*. synthesized Co_3_O_4_ with exposed {110} planes for oxygen evaluation reaction (OER) and discovered that Co^3+^ was the main active site that accelerated the reaction^24^. Dai *et al*.^18^ investigated the active reaction sites and revealed that the catalytic activity of the hybrid for ORR was significantly improved by N doping of mGO. Choi *et al*. designed spinel-type ZnCo_2_O_4_ hybrid as efficient electrode for OER and found that the hybrid was more electroactive than single Co_3_O_4_; hence, Co^2+^ cations played negligible roles in OER electrocatalysis^25^. Furthermore, XANES observations indicated that Co^3+^ was mainly active site for OER^26^.

Xiao *et al*. reported that the Li-ion storage performance of Co_3_O_4_ nano-octahedrons with the {111} plane outperforms that of nanocubes with the {001} plane^27^. Huang *et al*. further investigated the facet-dependent electrochemical properties of Co_3_O_4_ for heavy metal ions; the results indicated the higher sensitivity of Co_3_O_4_ nanoplates with exposed {111} facets than that of Co_3_O_4_ nanocubes with exposed {001} facets^28^. To achieve impressive electrocatalytic performance, scholars have focused on probing the influence of active sites in the Co_3_O_4_ structure on ORR and OER electrocatalysis. Nevertheless, no conclusive evidence is available regarding surface-dependent activity. Moreover, the underlying reaction mechanism must be elucidated.

In this study, we report a simple one-pot synthesis protocol that offers control over homogenously anisotropic crystal growth of N-GO/Co_3_O_4_ nanocrystals in hierarchal architectures of cuboid rectangular prism-shaped NRs and truncated polyhedrons. The genuine facet-dependent truncated polyhedron may render the catalyst surfaces to have dense-exposed, multi-facet sites along {311} and {111} planes and then develop a mat-like tangle of stable surface patterns for vast-range accessibility to active sites, and create catalyst surfaces with low adsorption energies and dense electron configuration during the oxygen reduction reaction (ORR). The superior electrocatalytic ORR activity of the N-GO/Co_3_O_4_ polyhedron nanocrystals in terms of electrochemical selectivity, durability and stability compared with NRs or commercial Pt/C catalysts. This finding indicates the synergetic contribution of polyhedron nanocrystals with high index, multi-functional {111} and {311} exposure facets, reactive Co^2+^ sites in both inner/upper zone top-on-plane surfaces, and a hyper electron-dense-cloud location are key components in improving the O_2_ adsorption and diffusion, and fast kinetics of electrons. These results strongly confirm the superior electrochemical stability of N-GO/Co_3_O_4_ polyhedrons catalyst, which is an indispensable feature for high -performance energy systems.

## Results and Discussion

**Figure 1:**
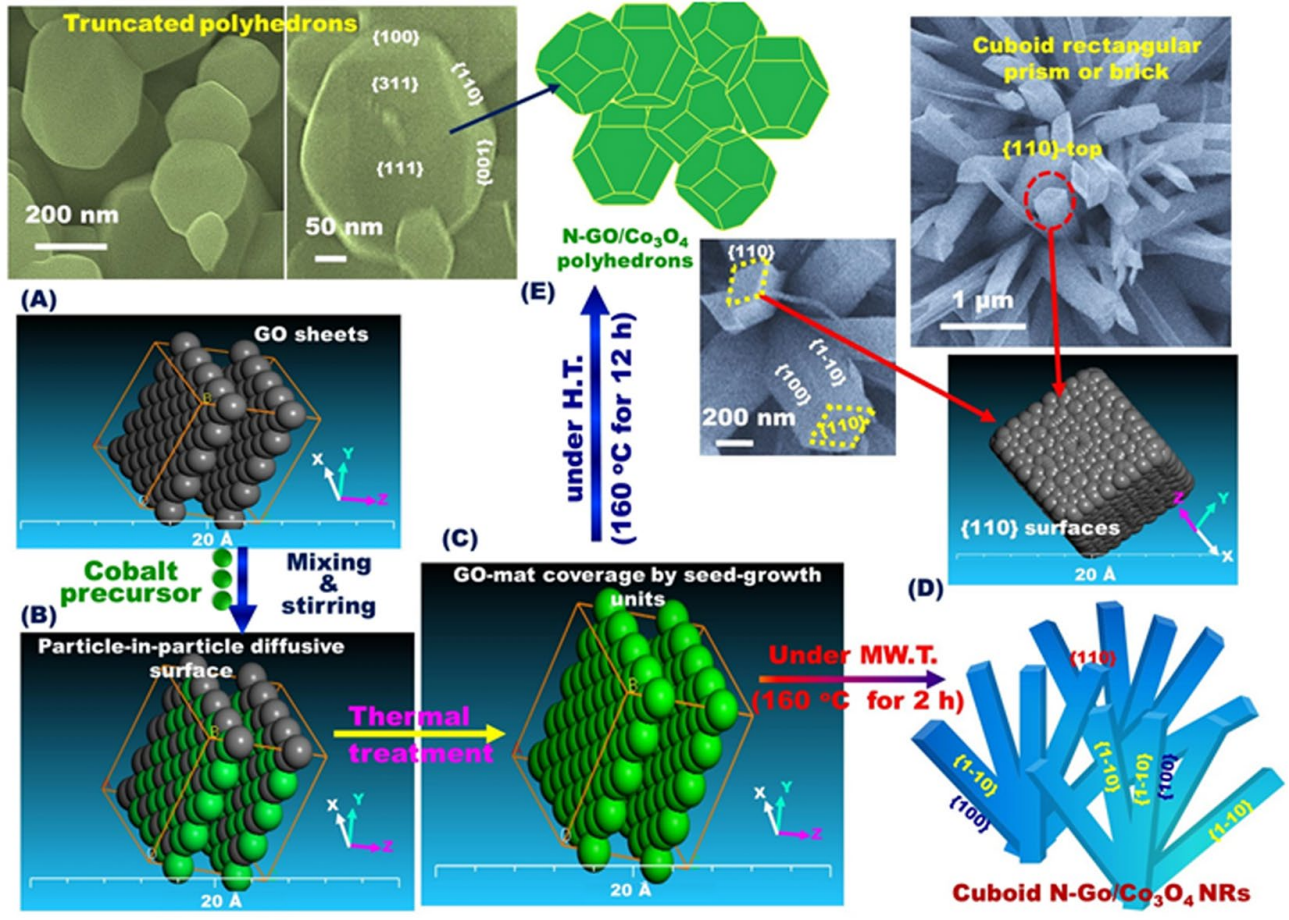
(**A**–**C**) Schematic illustration showing homogenously self-propelled diffusion method of heterogeneous N-GO/Co^2+^-ions/NaOH/urea (surfactant-free) composite domains was applied to control engineering unique single crystal N-GO/Co_3_O_4_ NRs and polyhedrons with abundance exposure of outstandingly high energy crystal planes (**D**,**E**). The surface engineering of truncated N-GO/Co_3_O_4_ polyhedrons oriented along multi-functional surface facets (such as {311}, and {111}) dominants) (**E**). The surface engineering of the N-GO/Co_3_O_4_ NRs was oriented along {110}-plane spreading out vertically from the core/orb of NR band-like vases to the top-surfaces in cuboid rectangular prism or brick without defects or distortion (**D**). The anisotropic growth of polyhedron and NR architectures was achieved through time-dependent hydrothermal (H.T.) and microwave (MW.T.) treatments, respectively. Engineering of homogenously self-propelled diffusion steps of N-GO/Co_3_O_4_ polyhedron and NR crystal structures were calculated through two layers of GO by density functional theory (DFT).

A simple synthesis based on homogenously self-propelled diffusion of heterogeneous N-GO/Co^2+^-ions/NaOH/urea (surfactant-free) composition domains was applied to control engineering of unique N-GO/Co_3_O_4_ nanocrystal structures, axially hierarchal NRs, preferential nanocrystal polyhedron orientations, multi-exposed crystal {311}, {111}, and {110} facet surfaces using one-pot protocol. As Shown in Fig. 1 and S1, the building of N-GO/Co_3_O_4_ hierarchal structures through self-propelled ion-to-ion diffusion pathways associated with particle-in-particle morphological shape growth of phase composition domains can be developed by time-dependent (i) stirring-, (ii) microwave/hydrothermal-, and (iii) annealing-assisted synthesis (see experimental section). The mechanistic growth of nanocrystals through homogenously self-propelled diffusion of distributed ions/particles/seeds of cobalt precursor into GO domains may occur consecutively via four steps. The first step is that the surface intercalation between cobalt and GO sheets was occurred through the Co^2+^-ions immigration to intercalate with the oxygen- functional groups of GO sheet through electrostatic interaction. The second step is the particle-in-particle diffusive surface interaction (Fig. 1B). The GO sheets were diffusively imbedded between parallel layers of cobalt and form complementary binding interactions with Co^2+^ ions through balanced thermodynamic and kinetic processes. The third step is the formation of stable-centered Co(OH)_x_(CO_3_)_0.5_∙0.11H_2_O seed-growth units into GO mat cavities (Fig. 1C). After prolonged time of stirring at 60 °C, the intermolecular spacing enhances the movement velocity and diffusion between Co^2+^ nuclei-site/GO. As a result of seed aggregation and folding, the cobalt seeds attach and cover the GO mats, forming sandwich like-structures as stable seed-growth step of crystal units. The fourth step is the homogenous cluster-building growth in single crystals with time-dependent hydrothermal (H.T.) and microwave (MW.T.) conditions. The seed growth unit coalesces and forms clusters during aggregation. Under H.T and MW.T conditions, the potentially anisotropic crystal growth might direct the aggregated unit cluster blocks to stabilize the high-energy surface of the preferentially arranged shape morphology and crystal geometry of nanorods and polyhedrons under time-dependent microwave (MW.T.) and hydrothermal (H.T.) conditions, respectively (Fig. 1D,E). Finally, the high-temperature annealing under N_2_ gas flow led to the phase transition of GO/Co(OH)_x_(CO_3_)_0.5_∙0.11H_2_O to thermally-stable, mesoscopic N-doped-GO/Co_3_O_4_ polyhedron and NR single crystals with relatively high surface area (≈50 m^2^/g) and mesopore space cavity of ≈20 nm (see Supporting Information, Figs S2 and S3)^26,27^. Fig. 1 show evidence that the homogenously anisotropic diffusion, interaction and growth of the crystal leads to produced polyhedron with newly multi-functional surface facets such as {311} and {111} dominants, and with highly truncated morphology, for the first time. Second, the randomly distributed and densely tilting-tangles along the vertical band networks of NR patterns led to create internal effects and voids (Fig. S3). This internal channels may then be produced continuous electron parallel to the c-axial orientation along {110} plane.

**Figure 2:**
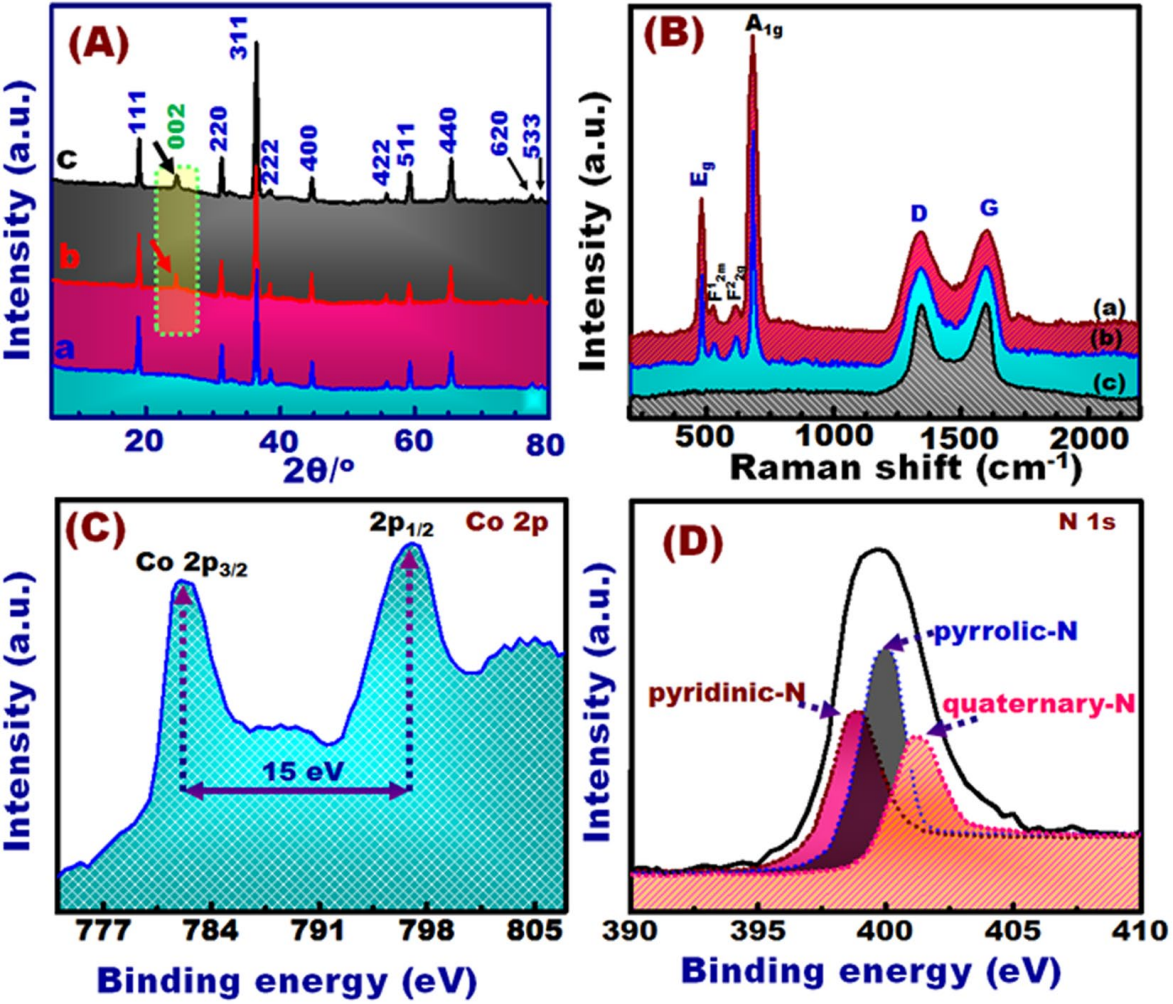
(**A**) WA-XRD spectra of N-GO/Co_3_O_4_ polyhedrons (a), N-GO/Co_3_O_4_ NRs (b), and Co3O4 polyhedrons. (**B**) Raman spectra of the N-GO/Co_3_O_4_ polyhedrons (a), N-GO/Co_3_O_4_ NRs (b), and N-GO (c). (**C**,**D**) High resolution XPS spectra of N-GO/Co_3_O_4_ polyhedrons with Co 2p (C) and N 1 S (**D**), respectively.

The phase purity of Co_3_O_4_single nanocrystals with N-GO/Co_3_O_4_ composite catalysts was supported by WA-XRD analyses as depicted in Fig. 2A. All diffraction patterns indicated that the face-centered cubic (fcc) phase of Co_3_O_4_ (JCPDS 42–1467) can be formed in pure single crystal with the formation of N-GO/Co_3_O_4_ NRs or polyhedrons^23^. The weak diffraction peak observed at 24.2° is mainly ascribed to the (002) reflection of carbon (graphene source). The Raman spectroscopies of N-GO/Co_3_O_4_ composite NR and Polyhedron single crystals shows real evidence of the engineering of N-graphene-diffused into Co_3_O_4_ single crystal structures through homogeneously particle-in-particle morphological shape growth of heterogeneously organometallic composition domains (Fig. 2B). Four evident peaks were located at approximately 480, 525, 615, and 682 cm^−1^ which correspond to E_g_, F^1^_2m_, F^2^_2g_, and A_1g_ modes of Co_3_O_4_, respectively^28^. In addition, the distinctive peaks observed at 1345 and 1600 cm^−1^ are mainly due to the characteristic D– and G–bands of the graphene component, respectively^29,30^. The D-band refers to the structural defects (A_1g_ symmetry), whereas the G−band represents the in-plane bond-vibration (sp^2^) of carbon atoms (Fig. 2B).

XPS measurements were performed to illustrate the compositions and chemical status of N-GO/Co_3_O_4_ polyhedrons as illustrated in Figs 2C,D and S4. The wide survey XPS spectrum exhibited four sharp peaks located at 284.6, 400, 530.1, and 780.1 eV corresponding to the characteristic features of C 1 s, N 1 s, O 1 s, and Co2p of carbon, nitrogen, oxygen, and cobalt elements of the investigated specimen, respectively (Fig. S4). The high -resolution Co2p spectrum results presented two major peaks at 782 and 797 eV that matched with the low energy band (Co 2p_3/2_) and high energy band (2p_1/2_) spin–orbit peaks of Co^2+^ ^31^. The energy difference between the peaks (Fig. 2C) is nearly 14 eV and is in good agreement with the literature and further demonstrates the presence of both Co^2+^/Co^3+^ species in the NR or polyhedron structure^32–35^. In addition, the weak 2p satellite features observed at binding energies at 788.5 and 803.3 eV indicate the formation of spinel structure, in which Co^2+^ cations conquer the tetrahedral sites and Co^3+^ cations occupy the octahedral sites in the in the crystal lattice^33^. Notably, the surface-adsorbed species can effectively reduce oxygen. The XPS analysis results of N-GO/Co_3_O_4_ polyhedrons and NR samples showed that the Co^3+^/Co^2+^ ratio of polyhedron (0.74) is lower than that of the nanorod structure (0.82).

Moreover, the N1s spectrum show its integration from three sub-peaks that appeared at 399.2, 401.0, and 403.3 eV binding energies due to pyridinic-N, pyrrolic-N, and quaternary-N, respectively^34,35^ (Fig. 2D). The quantitative analyses show that the nitrogen content in the polyhedron nanostructure determined deconvoluted high-resolution N 1 s spectra that reached approximately 5.18%. The corresponding fractions of each component of nitrogen were found to be 41.3%, 34.23%, and 23.47% for pyridinic, quaternary, and pyrrolic type, respectively. Interestingly, the ORR activity of electrocatalysts could be significantly affected by pyridinic and quaternary components of N than pyrrolic N^36–38^.

Together, the XRD, Raman, and XPS analyses provide strong evidence of the effectiveness of homogenously self-propelled diffusion associated with particle-in-particle growth mechanism, on which the novel, N-GO/Co_3_O_4_ NRs and polyhedrons were formed in one-pot synthesis with hierarchal engineering of (i) arrangement of heterogeneous N-GO/Co^2+^/Co^3+^/O^2−^ atomic sites in NR or polyhedron nanocrystals, (ii) preferential polyhedron nanocrystal-sized orientations with truncated morphology, multi-functional, high-index exposure crystal {311}, {111}, {100} or {110} facets (Fig. 1), and (iii) building of one-dimensional, heterogeneous (N-GO/Co_3_O_4_) composites in NR nanocrystals in band-like vases spreading out from the core of their orb and along preferential *c*-axial direction. These features in N-GO/Co_3_O_4_ catalysts may attain low adsorption energy, and dense, well-distributed electron configuration on multi-exposed surface facets and around the catalytically active Co^2+^ sites, as an avenue to design of electrode nano-pattern energy devices.

**Figure 3:**
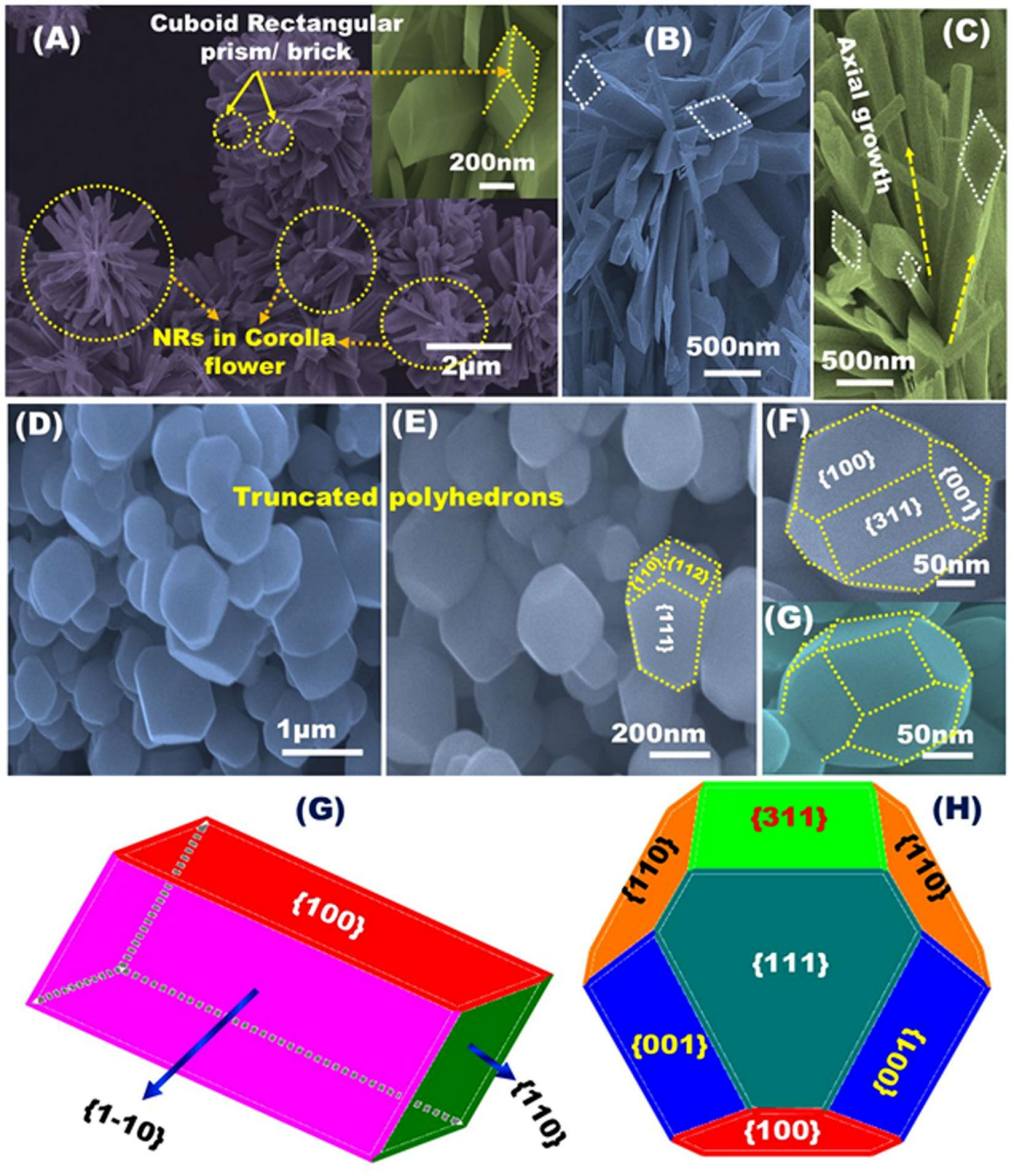
(**A**–**H**) Top-view SEM observations of N-GO/Co_3_O_4_ composite nanocrystals and their corresponding models indicting the existence of high exposure facets. (**A**–**C**) SEM images of N-GO/Co_3_O_4_ NRs and (**D**–**F**) SEM images of N-GO/Co_3_O_4_ polyhedrons. (**G**) Optimized shape-dependent model of NR structure in cuboid rectangular prism or brick, and (**H**) suggested model of polyhedron structure with highly truncated morphology.

The top-view field emission scanning electron microscopy (FE-SEM) micrographs show evidence of well-grown axial NR bands in corolla of a flower with a predominance of exposed regular, sharp, and smoothly flatten surfaces (Fig. 3A–C). The NRs have a length of sub-micrometers and a width of approximately 250–350 nm, which energetically favors axial branching-like bands ended with rectangular-shaped archery at the top-surfaces (Fig. 3A–C). The NR are mainly consisting of cuboid rectangular prism or brick crystals grown along preferentially exposed-breadth {110} dominant planes, with an axial-lengthy direction of {1–10} and its opposite {100} planes. Those three plane sets are mutually perpendicular (Fig. 3G). This results elucidate that the fabrication strategy offers control over the anisotropic crystal growth of N-GO/Co_3_O_4_ NRs in cuboid rectangular prism morphology along {110}-plane that spreads out vertically from the core/orb of NR band-like vases to the exposed rectangular-top-surfaces without defects or distortion, for the first time. Furthermore, the dense {110}-orientation from the core NR bands-like vases in the same direction of axial-lengthy {1–10} plane may act as a vascular system for the transport and retention of captured O_2_ molecule interiorly for longer time.

Figure 3D−F displays FE-SEM images of N-GO/Co_3_O_4_ polyhedron structure with highly truncated morphology. The polyhedrons were grown along preferentially exposure, highly reactive and multiple functional crystal facets. The size of the polyhedron particles is approximately 200–400 nm and is dominated by highly reactive crystals with tunable number of thermodynamically stable exposed planes of low-and-high index facets {110}, {311}, and {111} dominates with the existence of non-dominant {001} and {100} facets (Fig. 3H). With the formation of NRs and polyhedrons, the micrograph shows no distinct evidence of an extended continuous network of GO mats, attributing to the complementary binding interactions between the GO and Co^2+^ during the situ crystal growth through homogenously self-propelled diffusion method.

**Figure 4:**
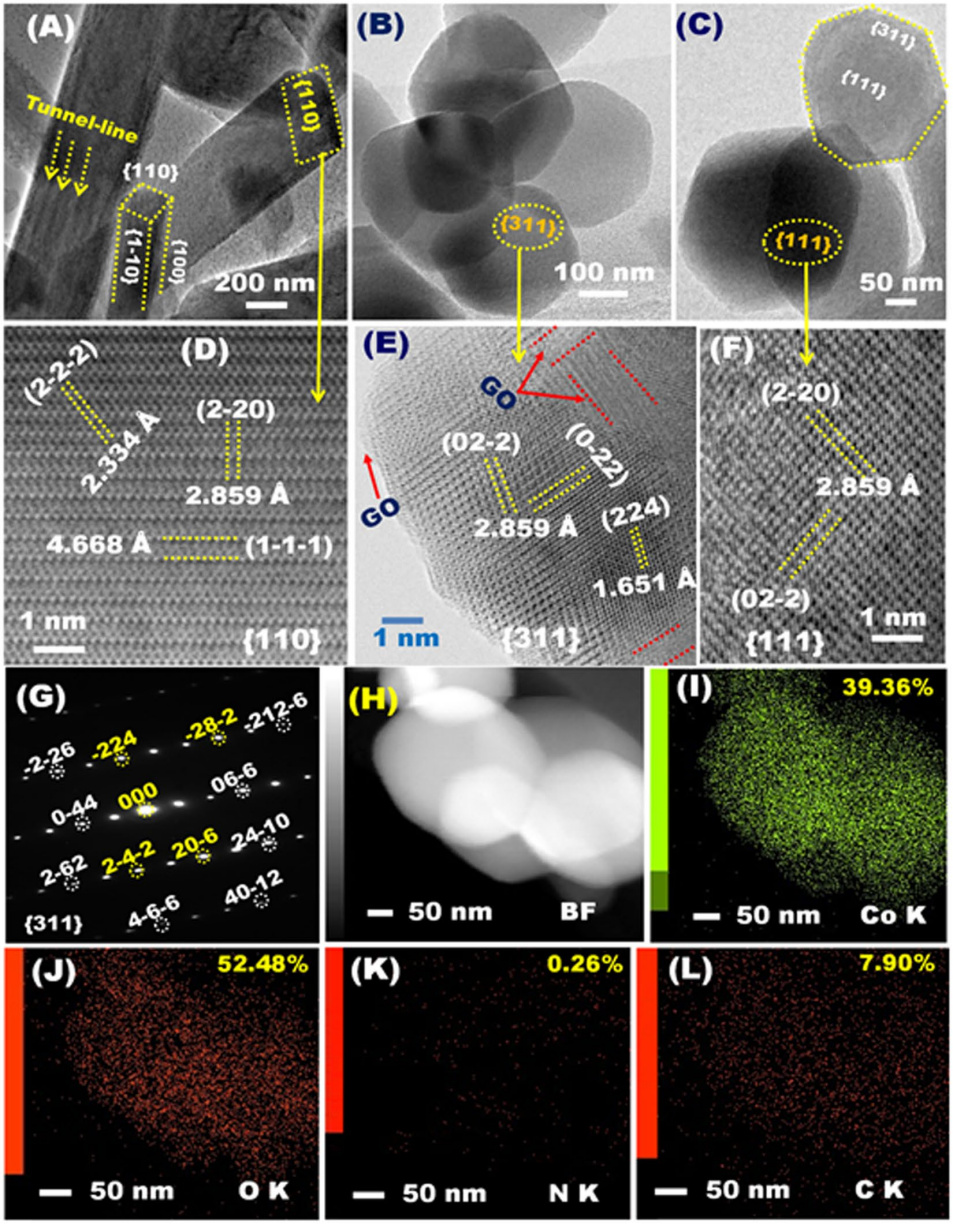
(**A**–**L**) HAADF/STEM micrographs of N-GO/Co_3_O_4_ nanocrystals at different locations. (**A**) Low magnification HAADF/STEM image of N-GO/Co_3_O_4_ NRs, (**B**,**C**) Low magnification HAADF/STEM image of N-GO/Co_3_O_4_ polyhedrons, (**D**) high magnification HAADF/STEM image of N-GO/Co_3_O_4_ NRs along {110} plane, and (**E**,**F**) high magnification HAADF/STEM image of N-GO/Co_3_O_4_ polyhedrons down {311} and (111) planes. (**G**) The corresponding STEM/ED micrograph N-GO/Co_3_O_4_ polyhedrons recorded along {311} crystal plane. (**H**) Bright field (BF)/STEM image of truncated polyhedron grown along preferentially exposure, highly reactive and multiple functional crystal facets. (**I**–**L**) STEM-EDS mapping analyses and the elemental composition contents of N-GO/Co_3_O_4_ polyhedrons; (**I**) Cobalt, (**J**) oxygen, (**K**) nitrogen, and (**L**) carbon mapping.

Side-view high angular annular dark-field (HAADF)–scanning/transmission electron microscope (STEM) patterns (Fig. 4A,D) revealed NR structures that had extended vertically in cuboid rectangular prism or brick shapes from the core of c-axis and had oriented along {110} plane. The {110}-prismatic rectangular edge scale of the NRs might typically measure about 250–400 nm breadth. The high-magnification HAADF-STEM image of NRs indicates the anisotropic growth of the rectangular-shaped top-surfaces along {110} crystal plane, as evidenced from the observation of dominant (1−1–1) and high-index (2–20), and (2-2-2) lattice planes (Fig. 4D). HAADF–STEM image of the NRs that grown with an axial-lengthy direction of {1–10} plane shows visual tunnel-line channels (see arrows, Fig. 4A), leading to high electron density movement during ORR.

Top-view HAADF-STEM images of truncated polyhedrons indicates that the N-GO/Co_3_O_4_ nanocrystals are grown along multi-functional surface facets, and primarily oriented along hexagon {111} and high-index {311} dominant planes (Fig. 4B,C). The high-resolution (HR) HAADF-STEM and the corresponding ED patterns (Fig. 4E–G) show abundantly aligned facets exposed along the preferential orientation {311} and {111} planes. HR- HAADF-STEM image (Fig. 4F) shows well-aligned, matched d-spacing lattice plane with 2.859 Å along the dominant {111} crystal plane, indicating the anisotropic nanocrystal growth of polyhedron along hexagon {111} facet. The ED-STEM image (Fig. 4G) further revealed abundant resolutions around the {311}-principle zone-dominant plane with high-index lattice planes and dense-line spots, for example, of (0–44), (06-6), or (2-4-2), (20-6), (-28-2), (-2-26), 24-10), (212-6) or (−4−6-6), and (40-12) fringes, indicating the structure of single crystal of N-GO/Co_3_O_4_ truncated polyhedron. This genuine facet-dependent polyhedron may render the catalyst surfaces to have dense-exposed, multi-facet sites and then develop a mat-like tangle of stable surface patterns for vast-range accessibility to active sites, and create catalyst surfaces with low adsorption, and binding energies.

Significantly, Fig. 4E shows that nanoscale (2 nm-breadth) prickle-like ridges and hocked cavities were marginally covered the {311}-plane edges of the polyhedron, leading to decrease the s binding energy of O_2_ molecules onto surfaces, and enhance dense location of active Co^2+^ sites on the upper-zone top-on-plane (Fig. 4E). The HR-HAADF-STEM image shows well-distributed GO mat as grass-skin along all edge and bulk surface facets, indicating the effective of synthesis method to well-controlled, homogenous diffusion of GO into Co_3_O_4_nanocrsytals (Fig. 4E). The N-GO blade forming grassy surface mats at the edged scale ridges of {311}-N-GO/Co_3_O_4_ (see Arrow, Fig. 4E) leads markedly to captured O_2_ molecules and sustained relaxation of the active site Co^2+^ atoms after 3000 times reuse/cycles of ORR.

Figure 4H–L shows BF-STEM image of pronounced multi-exposed surfaces of N-GO/Co_3_O_4_ polyhedrons and the corresponding elemental mapping results with the atomic configuration and distribution of Co, O, N, and C elements within (i) the truncated polyhedron architectures and (ii) crystal surface facets. The STEM-EDS images exhibited a marked atom-to-atom anisotropic growth geometry of truncated polyhedrons with well-distributed atomic surface active sites along all exposed low- and high-index planes. Increasing the number of topographic facets, well-distribution of actively atomic sites in polyhedron or NR particle-like islands are key to design catalytic electrodes for ORR.

**Figure 5:**
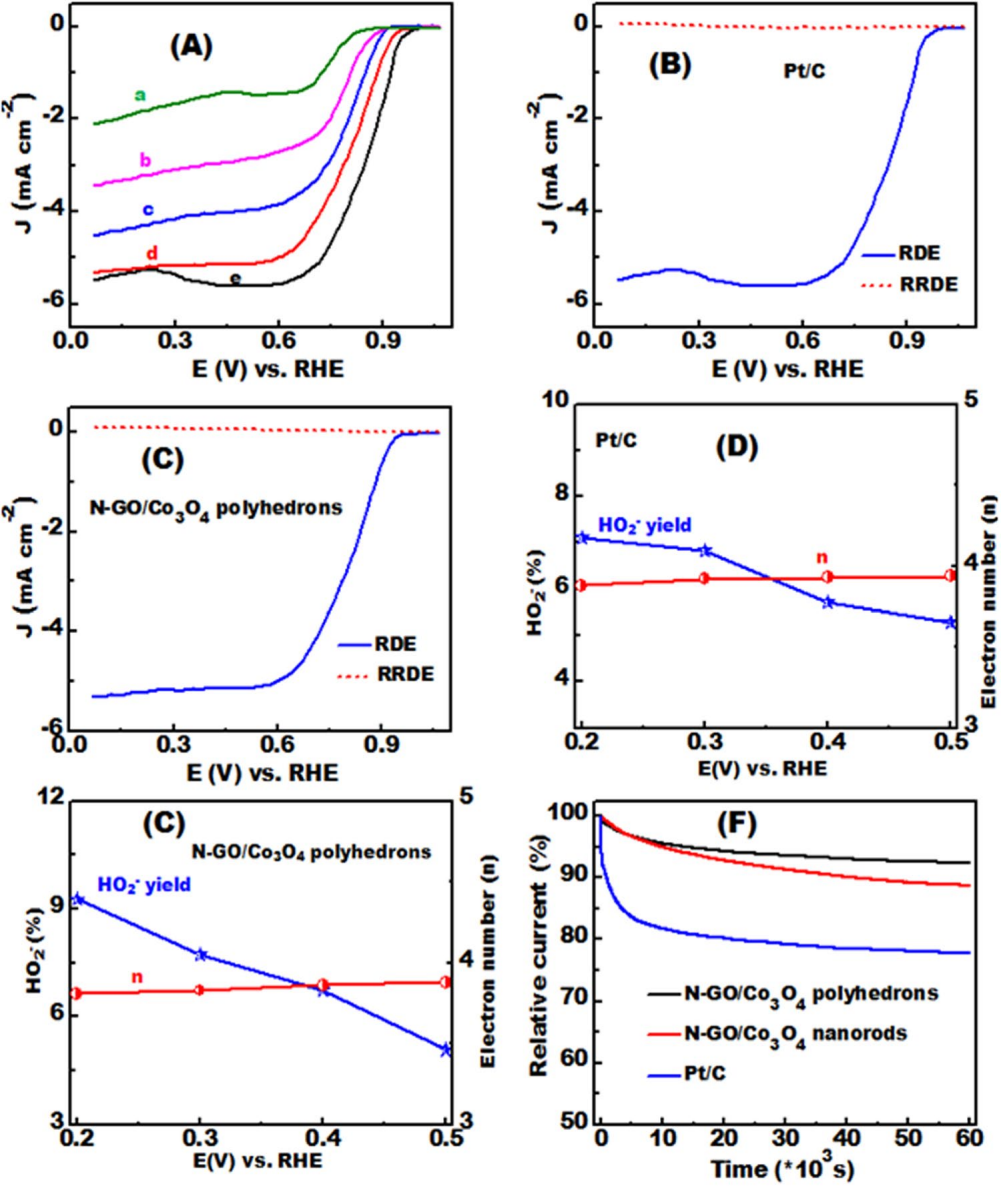
The ORR polarization curves (**A**), collected in 0.1 M KOH O_2_-saturated solution at 1600 rpm and at room temperature for (a) N-GO, (b) N-Co_3_O_4_, (c) N-GO/Co_3_O_4_ NRs, (d) N-GO/Co_3_O_4_ polyhedrons, and (e) commercial Pt/C catalysts. RDE and RRDE measurements of the ORR characterizations in terms of the electron transfer number (n) and the hydrogen peroxide content (HO_2_^−^) given in % collected in 0.1 M KOH O_2_-saturated solution at N-GO/Co_3_O_4_ hybrid polyhedrons (**B**,**D**), and commercial Pt/C catalyst (**C**,**E**). Current-time relationships (**F**) obtained by chronoamperometry test at a fixed potential of −0.2 V (*vs*. Hg/HgO) at N-GO/Co_3_O_4_ polyhedrons and commercial Pt/C catalysts in O_2_-saturated 0.1 M KOH solution.

The electrocatalytic performance of N-GO/Co_3_O_4_ hybrid nanocrystals and commercially available Pt/C catalysts for ORR was initially evaluated by rotating disc electrode (RDE) measurements in O_2_-saturated KOH (0.1 M) at 1600 rpm under similar catalyst loadings. In O_2_-saturated solution, both nanorods and polyhedrons, such as N-GO/Co_3_O_4_ hybrids (Fig. 5A, curves c and d), show higher activities than bare Co_3_O_4_ polyhedrons, as demonstrated by their current densities. When GO was incorporated into Co_3_O_4_, the hybrids exhibit large cathodic currents. This finding indicates that the enhanced electrochemically electroactive surfaces of GO promoted Co_3_O_4_. The enhanced ORR electroactivity of the N-GO/Co_3_O_4_ hybrids indicates that cobalt species synergistically coordinate with graphene matrix and nitrogen functionalities rather than stand alone as Co_3_O_4_. In addition, Co–N bonds play a vital role in increasing the electron density on the surface Co atoms of the N-GO/Co_3_O_4_ hybrids, thereby enhancing their electrochemical properties.

The presence of numerous surface groups on the GO sheets enhances the valence state of the metal ions, leading to rapid reaction kinetics. The embedding of GO into the hierarchal structure introduces atomic charge density, which enables facile charge transfer from the carbon matrices to the adsorbed oxygen molecules and subsequent formation of superoxide ions. Consequently, the O-O bond weakens, and the ORR activity improves.

This finding demonstrate the synergetic role of N-graphene-promoted Co_3_O_4_ nanocrystals to boost (i) the integrative GO mats with high in-plane conductivity that acts as an electronic trigger for shuttling electrons and (ii) N-heteroatoms in nanocrystals are more beneficial for enhancing the ORR catalytic performance of the electrocatalysts. As shown in Fig. 5A, the cathodic current density at 0.05 V (*vs*. RHE) of the investigated catalysts follow the order N-GO/Co_3_O_4_ polyhedrons (curve-d) > N-GO/Co_3_O_4_ NRs (curve-c) > Co_3_O_4_ polyhedrons (curve-b) > N-GO (curve-a). Accordingly, the current density in the linear sweep voltammograms is dependent on the accessibility of the active sites for ORR. The onset potential confirms the intrinsic features of the catalytically active sites (Table S1).

Additionally, the ORR onset potential and half-wave potential of the N-GO/Co_3_O_4_ polyhedrons (0.91 and 0.81 V, respectively) are lower than those of commercial Pt/C (0.94 and 0.84 V, respectively) (Fig. 4A, curve e) but relatively higher than those of the N-GO/Co_3_O_4_ nanorods (0.88 and 0.78 V, respectively), Co_3_O_4_ polyhedrons (0.85 and 0.76 V, respectively), and N-GO (0.81 and 0.72 V, respectively). The intrinsic ORR activity of the N-GO/Co_3_O_4_ polyhedron catalysts with close-matching features to that of commercially available Pt/C catalyst is mainly attributed to massive exposed surface, and high-index active site planes of truncated polyhedrons may lead to a vast-range accessibility of electron to pass along the top-charge Co^2+^-surfaces and also the in-depth center of the {111}, and {311} crystal planes. Therefore, the charge acceptance of the Co^2+^ species remarkably differs between the polyhedrons and nanorods.

ORR is achieved at electroactive sites associated with the oxidation state cations (Co^2+^) of the Co_3_O_4_ surfaces^20^. These Co^2+^ active sites serve as donor–acceptor reduction sites for Co_3_O_4_ by capturing electrons and consolidate the electronic configuration of the O_2_ molecules throughout ORR^39^. These reports suggested that Co^2+^ cations are the main active sites for ORR; moreover, the amount of Co^2+^ species formed on the N-GO/Co_3_O_4_ polyhedron surface is higher than that on the other catalysts. The electrocatalytic performance of the proposed catalysts is comparable with that of similar electrocatalysts reported previously.

A series of linear sweep voltammograms (LSVs) were performed at different rotating speeds (400–2000 rpm) in O_2_-saturated KOH (0.1 M) solution to determine the number of electrons transferred (*n*) and illustrate the ORR kinetics of N-GO/Co_3_O_4_ polyhedron nanocrystals and commercial Pt/C catalysts (Fig. S5A–D). Based on the collected LSV curves, the limiting current density of the catalysts increased with increasing rotation speed because of fast oxygen flux to the catalyst surface. The corresponding Koutecky−Levich (K−L) plots (J^−1^ vs. ω^−0.5^) within the potential range of 0.2–0.5 V versus RHE display good linearity of the fitted lines; hence, the reaction belongs to the first-order kinetics with respect to oxygen molecules (Fig. S5B,D). The number of electrons transferred (*n*) per O_2_ molecule involved in ORR was calculated according to Equations 1 and 2 (supporting information) based on J^−1^ versus ω^−0.5^ relationships^18,40^. The *n* for the N-GO/Co_3_O_4_ polyhedrons is 3.91, which is relatively close to that of the commercial Pt/C (*n* = 3.96).

The ORR at the N-GO/Co_3_O_4_ polyhedrons proceeds through direct four- electron pathway. These findings demonstrate the superior electrocatalytic ORR activity of the N-GO/Co_3_O_4_ polyhedron nanocrystals and further confirm the synergistic contribution of (i) multifunctional and active topographic facets, (ii) high-index, dense-exposed, and homogenously well-distributed active sites (island-like), and (iii) prickle-like ridges, hocked cavities, and N-GO blade of grass surfaces covered the {311}-plane edges of polyhedrons to activate the catalytic nature of the catalyst significantly.

RRDE measurements were performed in O_2_-saturated KOH (0.1 M) solution to determine the amount of hydrogen peroxide species (HO^2−^) formed during ORR process at the disk electrode for the N-GO/Co_3_O_4_ polyhedron catalyst (Fig. 5B–E). The RRDE analysis was further conducted to compare *n* values between N-GO/Co_3_O_4_ polyhedrons and Pt/C catalysts (Fig. S5B,C). The *n* values computed based on the analyzed disk and ring currents recorded at 1600 rpm in KOH (0.1 M) solution are 3.81−3.85 and 3.88−3.94 for N-GO/Co_3_O_4_ polyhedrons and Pt/C, respectively, over the potential range of 0.2–0.5 V versus RHE. Thus, the ORR on N-GO/Co_3_O_4_ polyhedrons proceeds through four-electron mechanisms similar to that catalyzed by Pt/C (Fig. S5D,E). As shown in Fig. S5C, the hybrid polyhedrons possess a small ring current due to HO^2−^ intermediates (hydrogen peroxide oxidation). According to the RRDE responses, the percentages of HO^2−^ species with respect to the oxygen reduction products are 9.27% and 7.12% for N-GO/Co_3_O_4_ polyhedrons and Pt/C, respectively (Fig. S5D,E). Our finding indicated that the H_2_O_2_ yield of the mesoporous N-GO/Co_3_O_4_ polyhedrons is lower than that of the MnCo_2_O_4_/N-doped graphene hybrid^25^. Hence, the mesoporous N-GO/Co_3_O_4_ polyhedrons hold considerable potential as energy technologies in the future.

The electrocatalytic durability is a significant factor that should be considered to develop the practical application of electrocatalysts. Continuous potential sweeps for 3000 cycles were performed in O_2_-saturated KOH (0.1 M) solution at room temperature under a rotation speed of 1600 rpm to compare the structural stability of the composite polyhedrons with that of the Pt/C catalyst (Fig. S6A,B). Under the same experimental conditions, the LSV measurements of the N-GO/Co_3_O_4_ polyhedrons before and after 3000 cycles (Fig. S6A) exhibit remarkable long-term structure stability compared with that of Pt/C, indicating the remarkable cyclability and reproducibility of the polyhedrons. The N-GO/Co_3_O_4_ polyhedrons display a small activity loss with negative shift in the E_1/2_ of 15 mV for an extended period. For the commercially available Pt/C, the E_1/2_ decreases by about 32 mV (Fig. S6B) after continuous potential scan, despite the more reactive property of Pt than that of the metal oxides. Pt nanoparticles undergo severe aggregation to form large particles because of their high surface energy^40,41^. Moreover, the weak contact of the Pt nanoparticles to the carbon support can substantially decrease the ORR activity of Pt^40^. Hence, we can conclude that the synthesized N-GO/Co_3_O_4_ polyhedrons possess superior structural stability with efficient retention of electrocatalytic activity. The improved ORR performance of the N-GO/Co_3_O_4_ polyhedrons can be ascribed to the (i) synergistic interaction between the Co species and GO surface groups, leading to distinct structural stability and fast charge mobility at the catalyst/electrolyte interfaces and facilitating the reaction pathway; and (ii) abundance of electroactive sites, which directly modify the relative positioning of the conduction bands and improves the electronic configuration of the active catalyst, thereby increasing the catalytic efficiency.

The durability of the as-synthesized catalysts and commercial Pt/C was assessed and compared with the chronoamperometric spectra at + 0.8 V versus RHF in an O_2_-saturated KOH (0.1 M) solution by using a rotating disk electrode to confirm the superior electrochemical performance of the N-GO/Co_3_O_4_ hybrids. As shown in Fig. 5F, the ORR current densities of the as-grown N-GO/Co_3_O_4_ polyhedrons and nanorods are 7.58% and 11.25% degradation, respectively, over 60,000 s of continuous operation. By contrast, the commercial Pt/C electrode shows remarkably decreased voltammetric current by about 22.32% under the same testing conditions relative to its initial activity; this finding indicates the poor stability of the Pt/C electrode. The electrochemical stability of the N-GO/Co_3_O_4_ catalysts is significantly enhanced, and the as-grown nanostructures favor the stability, an indispensable feature for high-performance energy systems^42,43^.

Crossover influences due to methanol and stability are critical factors that affect the ORR electrocatalyst performance in fuel cells. To assess the tolerance of the N-GO/Co_3_O_4_ polyhedrons and Pt/C catalyst to methanol crossover, we mixed methanol (3 M) to a KOH (0.1 M) solution in the testing cell in the presence of oxygen. The obtained chronoamperometric signals are illustrated in Fig. S7. Thus, the chronoamperometric responses of the proposed catalyst and the commercial Pt/C catalyst show no obvious changes in the investigated N-GO/Co_3_O_4_ polyhedrons upon methanol introduction. This finding indicates improved methanol tolerance. By contrast, the observed chronoamperometric responses of the commercial Pt/C catalyst evidently decrease, indicating weak methanol tolerance.

Generally, oxygen reduction in alkaline media is a sophisticated electrocatalytic reaction due to the formation of various species and intermediates (i.e., O, OH, O_2_^−^, and HO^−^_2_). Tafel plots in the intermediate current density region were measured using kinetic currents from the steady-state polarization according to K−L equation to examine the catalytic kinetics of ORR for the composite catalysts. The representative Tafel plots of ORR for the N-GO/Co_3_O_4_ polyhedron catalyst and other controls are displayed in Fig. S8. The observed Tafel slopes of the N-GO/Co_3_O_4_ hybrids are 72.6 and 83.4 mV dec^−1^ for the polyhedron and NR, respectively; these values are lower than that of Pt/C (94.2 mV dec^−1^), demonstrating the high inherent catalytic activity of the as-synthesized hybrids toward ORR. Moreover, the Tafel plots of single GO and Co_3_O_4_ polyhedrons are 115.8 and 101.3 mV dec^−1^, respectively. Theoretically, the Tafel plot close to 59 mV dec^−1^ is attributed to the adsorbed oxygen intermediates, which obstruct surface coverage-dependent activation for electrocatalytic reactions. The results indicate that the oxygen reduction on the N-GO/Co_3_O_4_ polyhedron is correlated with the elimination of oxygen intermediates^39,44^.

**Figure 6:**
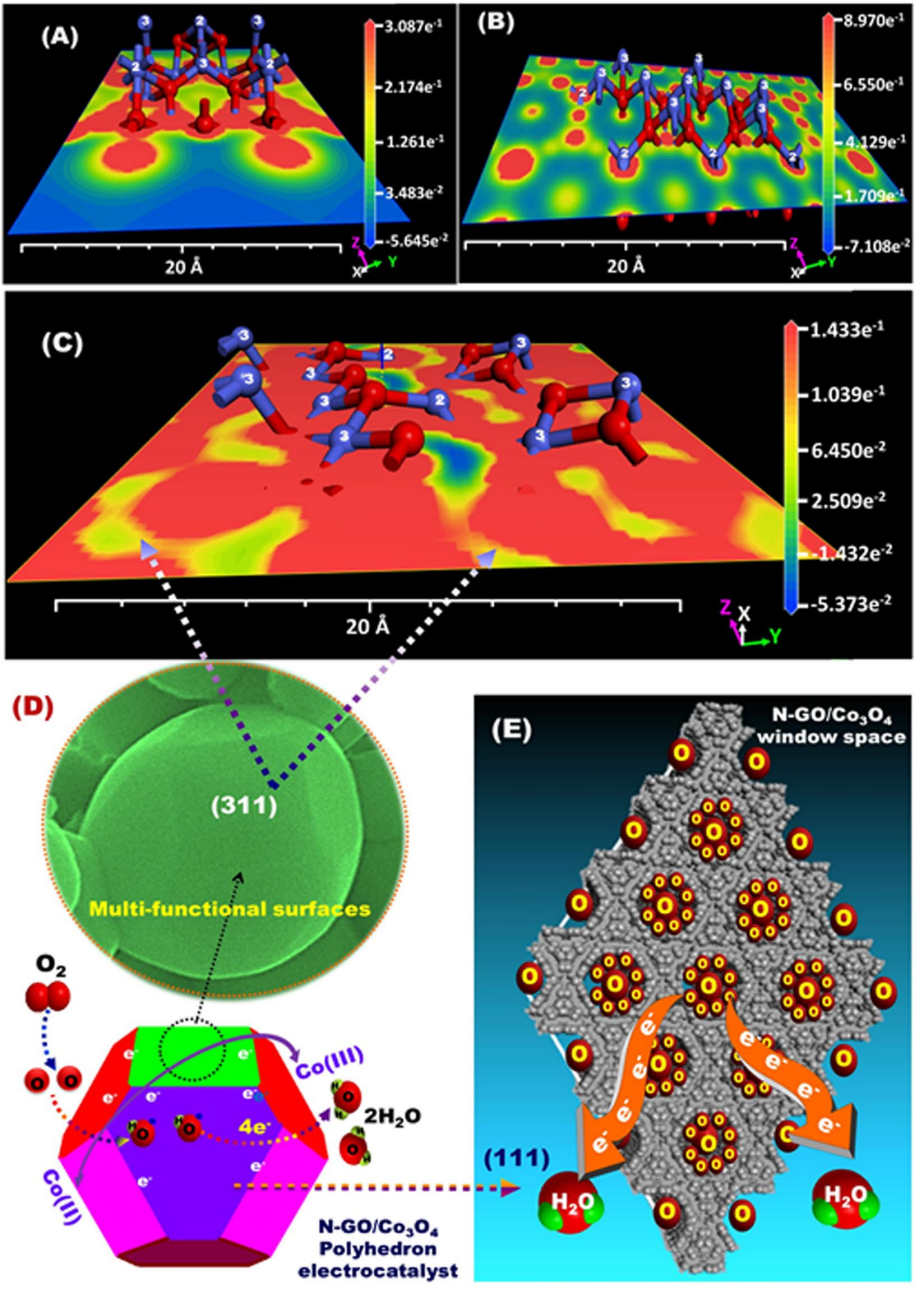
(**A**–**C**) Electrostatic potential energy maps (ESP-EM), surface potential configuration and charge distribution of Co^2+^, Co^3+^ (blue color) and O^2−^ (red color) atoms along the (**A**) {110}, (**B**) {111}, and (**C**) {311} crystal plane surfaces of NRs (A) and polyhedron (**B**,**C**) catalysts. (**D**,**E**) Representative illustration showing the mechanism of ORR onto the {311} plane surfaces (**D**) and into the {111} window mesopore spaces (**E**) of the N-GO/Co_3_O_4_ polyhedron crystal surfaces. The oxygen molecules were first adsorbed at the Co^2+^- active sites of exposed planes. The O_2_ molecule adsorbed perpendicularly to the actively catalytic-site surfaces and diffused into the pore space frameworks (**E**). The ORR was achieved via 4e- pathway (**D**,**E**). (D-top) FE-SEM microscope of N-GO/Co_3_O_4_ polyhedrons that primarily oriented along {311}-top-surface exposure plane. The ESP-EM calculations of the selected molecules were determined using BIOVIA Materials Studio.

To investigate the crystal facet-dependent ORR catalytic activity, as schematically illustrated in Figs (6 and S9), we quantitatively determined the charge surface density, electron distribution and configuration around the active surface Co^2+^/Co^3+^ sites, number of active-top-surface atoms, and adsorption surface energy along plane surfaces of {110}-NR, and {111}-, and {311}-polyhedron nanocrystal facets by DFT calculations. The distinct molecular design of nanocrystals for catalytic activity enhancement of the exposed surface sites is essential to improve the overall reaction kinetics. The densities of Co^3+^ and Co^2+^ active sites in the Co_3_O_4_ catalyst can provide good electrocatalytic activity toward ORR because the concentration of the catalytically active Co^2+^ sites on the surface of cobalt oxides positively influences the ORR reactivity. The spinel Co_3_O_4_ revealed surface-dependent catalysis owing to the change in Co^3+^/Co^2+^ percentages on the exposed surface facets^45–47^.

The ORR electroactivity of the as-synthesized cobalt oxides is sensitive to the number of Co^2+^ cations by controlling the growth mechanism of their morphologies. Many Co_3_O_4_ nanostructures are also enclosed by {111} and {110} planes. To the best of our knowledge, polyhedrons, such as Co_3_O_4_ nanostructures with {111} and {311} exposed facets, have not been reported yet.

More importantly, DFT calculations (Figs 6 and S9) shows first evidence that those three key factors of (i) the large number of the catalytically active Co^2+^ cations at the whole top surface layers of crystal, (ii) dense location of active Co^2+^ sites on the upper-zone top-on-plane exposure, and (iii) the electron density configuration and distribution around the Co^2+^ sites can favorably tune the electrochemical performances of the electrocatalyst for ORR. The configuration of surface-oxygen molecule adsorption on the first sublayer of Co^2+^ sites is more complex than that on the topmost surface layer. Such configuration resulted in volcano-shaped dependence based on the effect of the activation and desorption features of O_2_ molecules on the catalyst surface during ORR. Therefore, the location of Co^2+^ sites on various exposed planes significantly influences the catalytic activity toward ORR. Additionally, the corresponding strength of oxygen binding influences the ORR kinetics. The improved ORR activities of the Co_3_O_4_ nanocrystals might be associated with exposed facets and Co^2+^/Co^3+^ valence states formed by oxygen vacancies. On the {111} and {311} exposed surfaces, O_2_ is first selectively adopted on the subsurface oxygen vacancy due to the abundant negative charges. Consequently, the Co−O bond is formed. The adsorbed O_2_ captures the free electrons at oxygen vacancies and produces radical groups, which subsequently enhance the reduction of O_2_ through the four-electron pathway^48^.

In general, the O_2_ molecules are attracted to the catalytic active sites (Co^2+^) based on the Pauling mode. These active sites can efficiently transport electron to the absorbed O_2_ molecules^47^, which clearly indicates that the active Co^2+^ sites are catalytically active sites rather than Co^3+^ sites.

Furthermore, the valence species of metal oxides are considered catalytically active sites for activating adsorbed O_2_ and ion diffusion. Fast electron transport due to dense exposure of Co^2+^ sites also favors O_2_^−^ adsorption. The coexisting Co-O surface plays a synergistic role in breaking the O-O bond^49^. Therefore, fully covered surfaces with electron acceptors might be beneficial for O_2_^−^ adsorption. The Co-O shows coexisting Co_3_O_4_ {110} and {111}, and {311} might diminish the O_2_^−^ adsorption barrier on the active surfaces.

The Co^2+^ atom onto exposed {111} and {311} planes is preferentially oriented around the most-negative potential (electron clouds, red color) surfaces, leading to highly adsorbed oxygen molecule (O_2_) reactants. Significantly, with {111}- and {113}-polyhedron crystal planes, the Co^2+^ atom location was found in the dense electron-configuration and cloud-distribution on the upper-zone top-surfaces compared with {110} NR-top-surface exposures (Fig. 6A–C). As shown from electrostatic potential energy map (ESP-EM) patterns, the 2-Co^2+^-{311}-top-surface showed a hyper electron-dense-cloud location compared with the exposed 4-Co^2+^-{111}-top-surface along the multi-functional facets of polyhedron crystals.

The as-grown cobalt oxide clusters become more conductive than that of the semiconducting cobalt oxide clusters, thereby promoting facile electron transport from the valence band to the conduction band because Co^2+^ can more easily lose electrons than oxygen ions or other Co ions; hence, Co^2+^ is the active site. These features accelerate the electron mobility onto Co^2+^ centers, rate-determining processes, and ORR kinetics. In addition, the Co atoms are coordinated with the surrounding oxygen atoms at the top surface layers. The oxygen-rich Co_3_O_4_ {111} and {311} surfaces with fully covered active sites present high catalytic activity for ORR due to facile electron transport from O_2_^2−^ to the surfaces. Thus, the intermolecular charge transfer enables a net positive charge to enhance the ORR activity.

The catalytic mechanism allows the oxygen vacancies at the active top surfaces to significantly enhance the ORR activity^50–52^. The synergistic role of oxygen vacancies and exposed surfaces favors the improvement of the electrocatalytic performance for ORR. The DFT analysis indicates that the strong bonding between Co species and adsorbed O_2_ molecules on the generated oxygen vacancies could stretch the O-O bonds, leading to dissociation of O-O bonds. This finding is in agreement with that reported by Tompsett *et al*.^50^, who confirmed that the presence of oxygen vacancy enhances the catalytic activity of MnO_2_ toward ORR. Therefore, the generated oxygen vacancies are necessary for chemical reactions because they can strongly bind O_2_ molecules and facilitate their dissociation.

The DFT calculation indicates that the adsorption energy of oxygen molecules onto exposed polyhedron and NR crystal surface facets was determined to be approximately −5.9, −5.86, and −5.67 kcal/mol for {311}, {111}, and {110} planes, respectively. This finding indicates that ORR activity onto exposed surfaces increases in this trend {110} < {111} < {311} planes. Thus, the oxygen molecules will easily adsorb onto the active {311} plane surfaces and strongly bond due to the unsaturated dangling bonds leading to better reaction kinetics than that of {111} and {110} planes.

Figure S9 shows evidence that the upper-top-on-plane configuration of the single crystal NRs or polyhedrons is banged by catalytically active Co^2+^ sites that significantly affected oxygen molecular diffusion and adsorption, and the electron/charge transport along the reactive facets (see also Fig. 6A–C). The Co^2+^ density in the upper-zone top-surface planes decreases in the order of {311} > {110} ≥ {111}. The fully exposed Co^2+^ top surfaces of the {111}, {311}, and {110} planes show four, two, and three catalytic active Co^2+^ sites, respectively. Although the {111} facet affords a large number of active Co^2+^ sites in both inner-/upper-zone plan of top-surface layers, but the high-index{311}-surface catalysts exhibited the highest ORR reactivity, as evidenced from the adsorption energy of oxygen molecules onto {311} active sites. Polyhedrons, such as Co_3_O_4_ hybrid nanostructures with predominantly exposed {111}/{311} planes, possess significantly enhanced ORR electrocatalytic activity than that of the Co_3_O_4_ hybrid nanorods due to abundant Co^2+^ sites at the {111}/{311} reactive planes. Therefore, fast electron transfer from Co^2+^ sites can considerably promote the catalytic activity for ORR^20,24^. With its facet-mediated behavior, cobalt spinel positive ions with dense charges (Co^2+^) occupy holes generated by oxygen ions. Thus, the Co^2+^ ions can effectively participate in the ORR due to enhanced dissociative adsorption and charge mobility. This result indicates that the process proceeds with the aid of Co^2+^ ions due to its single e_g_ occupancy^53^.

Our DFT calculations show real evidence, for the first time that not only the large number of catalytically active Co^2+^ cations at the top surface layer but also the dense location of active Co^2+^ sites on the upper-zone top-on-plane exposure. Moreover, the electron density configuration and distribution around the Co^2+^ sites are important for effective ORR because they reduce the side reactions associated with carbon and electrolyte.

In general, the polyhedron nanocrystals with high index, multifunctional {111} and {311} exposure facets, reactive Co^2+^ sites in both inner/upper zone top-on-plane surfaces, and a hyper electron-dense-cloud location are key components in improving the O_2_ adsorption and diffusion and fast kinetics of electrons. The O_2_ molecules are efficiently absorbed in the active sites, and the Co atoms are transformed into their oxidized state. Accordingly, the induced charge at the catalytically active centers can evolve the chemisorption mode of O_2_ molecules. This reaction weakens the O-O bond and enhances the kinetics of ORR^54^. The ORR is a surface-structure-sensitive reaction on electrodes and is attributed to the accessible active sites corresponding to the cations on the catalyst surfaces. According to the DFT analysis, the exposed Co^2+^ cations are more accessible than Co^3+^, indicating that the Co^2+^ ions are determinant in catalyzing O_2_ molecules and enabling high efficiency, reducing ORR overpotential, and enhancing cycle stability^55,56^. Thus, for polyhedrons, such as N-GO/Co_3_O_4_ nanocrystals, the atomic binding energy of oxygen largely governs the electronic modification of Co^2+^ sites engendered by the construction of an interfacial heterostructure between cobalt oxide and GO^57^. Notably, the Co^3+^/Co^2+^ ratio measured by XPS observation is in agreement with the electrochemical results obtained for ORR. In addition to surface exposure, the Co^2+^ active species play a pivotal role in the catalytic performance for ORR.

In summary, one-pot synthesis strategy offered a potentially effective control for (i) homogeneously atomic orientations of mesoscopic N-GO/Co_3_O_4_ nanocrystals from heterogeneous composition (surfactant-free domain), (ii) preferential polyhedron nanocrystal-sized orientations with truncated morphology, multi-functional, high-index exposure crystal {311}, {111}, {100} or {110} facets, and (iii) building of one-dimensional, cuboid rectangular prism-shaped NR nanocrystals in band-like vases spreading out axially from the core of their orb to the exposed rectangular-top-surfaces in the same direction of axial-lengthy, tunnel-line channel {1–10} plane may act as a vascular system for the transport and retention of captured O_2_ molecule interiorly. These features in N-GO/Co_3_O_4_ architectures create effective catalysts with low adsorption energy, and dense, well-distributed electron configuration on multi-exposed surface facets and around the catalytically active Co^2+^ sites, as an avenue to design electrode nano-pattern for ORR. Significantly, with {111}- and {113}-polyhedron crystal planes, the Co^2+^ atom location was found in the dense electron-configuration and cloud-distribution on the upper-zone top-surfaces compared with {110} NR-top-surface exposures. As a result, the polyhedron catalysts show better ORR activity than that of NR nanocrystals.

In addition, the polyhedron nanocrystals revealed outstanding electrocatalytic ORR performance and superior stability comparable to commercial Pt/C. Results also demonstrate that polyhedrons can significantly catalyze oxygen molecules via a 4^−^ electron pathway with excellent methanol tolerance. In particular, the polyhedron nanostructures exhibit superior durability after 3000 continuous potential cycles comparable to commercially available Pt/C catalyst. The present study can serve as basis for future designing of cost-effective and efficient electrocatalysts as alternatives to Pt-based catalysts for practical application in fuel cells and other technological energy systems.

### Experimental section

Unique N-GO/Co_3_O_4_nanocrystals of cuboid rectangular prism-shaped NRs and truncated polyhedrons were fabricated by using homogenously self-propelled particle-in-particle diffusion, interaction and growth protocol under the peculiarity of heating treatment (Figs 1 and S1). The polyhedron and NR architectures were synthesized through one-pot, time-dependent hydrothermal (H.T.) and microwave (MW.T.) treatments, respectively. The N-enriched hierarchal surfaces were obtained by a simple high-temperature annealing process under N_2_ gas flow, as evidenced from STEM-EDS profile (Fig. 4K).

First, N-GO/Co_3_O_4_ nanocrystals of cuboid rectangular prism-shaped NRs were fabricated as follows: cobalt acetate tetrahydrate (Co(C_2_H_3_O_2_)_2_.4H_2_O, 5 mM) and urea (CH_4_N_2_O, 12 mM) were dissolved in 37 mL of DI-H_2_O with stirring until a clear solution was obtained. Approximately 60 mg of solid GO, which was fabricated from graphite powder (see supporting information), was added to the Co^2+^ ion/urea solution under stirring for 1 h. The mixture was vigorously stirred for 16 h at 60 °C to form a homogeneous mixture, and then transferred into a 75 mL Teflon-lined autoclave. The anisotropic crystal growth of N-GO/Co_3_O_4_ NRs was occurred by treating the homogenous mixture thermally at 160 °C for 2 h under microwave irradiation (600 W). After cooling to 25 °C, the solid yield was rinsed several times using DI-H_2_O and absolute ethanol for removing the unreacted and soluble impurities and was subsequently dried at 60 °C overnight.

Second, N-GO/Co_3_O_4_ nanocrystals of truncated polyhedrons were fabricated according to the typical synthesis as follows: 25 mM cobalt nitrate hexahydrate (Co(NO_3_)_2_·6H_2_O) and 6 mM NaOH were mixed under stirring in 60 mL of DI-H_2_O. Then, 60 mg of the as-prepared GO was immersed into the above solution under vigorous stirring for 16 h 60 °C to form a homogeneous mixture. The mixture was loaded into a Teflon-lined stainless steel autoclave and maintained at 180 °C for 8 h before cooling naturally to RT. The precursor was centrifuged, rinsed thoroughly with DI-H_2_Oand ethanol for removing the unreacted ions, and finally dried.

Third, the N-GO/Co_3_O_4_ NR and polyhedron catalysts were obtained by annealing the precursors in a programmable furnace under nitrogen gas flow at 550 °C for 4 h with ramping rate 5 °C min^−1^. The loading amount of GO in the N-GO/Co_3_O_4_ NR and polyhedron composites was approximately of 8.44 wt%, and 8.15 wt%, respectively. The resulting N-GO/Co_3_O_4_ NR and polyhedron catalysts were used for the electrochemical ORR (see supporting information).

## Electronic supplementary material

Supplementary Information
