# Supplementary material for: Anisotropic N-Graphene-diffused Co3O4 nanocrystals with dense upper-zone top-on-plane exposure facets as effective ORR electrocatalysts
Source: Sci Rep. 2018 Feb 27;8:3740. doi: 10.1038/s41598-018-21878-w (PMC5829235; doi:10.1038/s41598-018-21878-w)
Supplement: Supplementary file 1 — Supplementary Information [file 41598_2018_21878_MOESM1_ESM.pdf]

Anisotropic N-Graphene-diffused Co_3O_4 nanocrystals with dense upper-zone top-on-plane exposure facets as effective ORR electrocatalysts

Diab Hassen,[1] Mohamed A. Shenashen,[1] Abdullah R. El-Safty,[1] Ahmed Elmarakbi,[2] Sherif A. El-Safty,[1*]

[1]National Institute for Materials Science (NIMS), Research Center for Strategic Materials, 1-2-1 Sengen, Tsukuba-shi, Ibaraki-ken, 305-0047, Japan.
[2]Department of Computing, Engineering and Technology, University of Sunderland, Edinburgh Building, Chester Road, Sunderland, SR1 3SD, United Kingdom.

TEL: +81-29-859-2135
FAX: +81-29-859-2501
E-mail: sherif.elsafty@nims.go.jp

Experimental section

Materials and chemicals

Potassium permanganate ($KMnO_4$), cobalt acetate tetrahydrate ($Co(C_2H_3O_2)_2.4H_2O$), and cobalt nitrate hexahydrate ($Co(NO_3)_2{\cdot}6H_2O$) were purchased from Tokyo Chemical Industry Company (TCI), Ltd., Japan. Graphite powder and Nafion solution were obtained from Sigma-Aldrich Company Ltd., USA. Urea (CH_4N_2O) and sodium hydroxide (NaOH) were supplied by Wako Co., Ltd., Osaka, Japan. Hydrochloric acid (HCl), sulfuric acid (H_2SO_4), and hydrogen peroxide (H_2O_2) were bought from Nacalai Tesque Co., Japan.

Preparation of graphene oxide (GO)

TheGO was prepared using a slightly improved Hummer's method.[1] In a typical synthesis procedure, 5 g of graphite powder was well dispersed into 40 mL of concentrated H_2SO_4 under continuous stirring for 2 h at room temperature (RT). Then, 3 g of $KMnO_4$ was slowly added to the mixture under magnetic stirring for 3 h at 0 °C, followed by ultrasonication treatment for 5 h at 45 °C to form a homogeneous solution. After which, 200 mL of deionized water was mixed with the obtained reaction mixture, which was kept at 100 °C for 20 min. Subsequently, 80 mL of H_2O_2 and 40 mL of HCl were added while stirring. The resulting brownish precipitate was separated by centrifugation, repeatedly washed with deionized water, and allowed to dry at RT. Prior to further investigation, graphene oxide was ultrasonically treated for 45 min and vacuum-dried at 60 °C for 8 h.

Electrochemical ORR using N-GO/Co_3O_4 NR and polyhedron catalysts

Electrochemical measurements of the samples were carried out on a Zennium/ZAHNER (Elektrik GmbH & Co. KG) electrochemical analyzer. The analyses were collected by a three-electrode cell using a rotating disk electrode (RDE, 5 mm in diameter) modified with the active catalytic material which served as the working electrode. Platinum wire and mercury/mercury oxide (Hg/HgO) were used as the counter and reference electrodes, respectively. Prior to the investigation, the working electrode was initially polished with alumina powder, washed with Milli-Q water, gently sonicated in acetone solution, and then dried. The catalyst ink was prepared by dispersing 10 mg of the active catalyst in 5 mL ethanol under sonication for 40 min.

5 μL of the catalyst ink was dropped onto the active area of the pre-polished glassy carbon, followed by 5 μL Nafion solution (0.05 wt%). The as-prepared glassy carbon was carefully dried in open air to form a stable film of the catalyst. For comparison, commercial Pt/C (20 wt% Pt content, Johnson Matthey) was prepared using the same procedure described above. The linear sweep voltammograms (LSVs) of the catalysts were recorded in O_2-saturated 0.1 M KOH solution at 50 mVs^{-1} and all the potentials were referred to the Hg/HgO reference electrode. The ORR kinetics was evaluated at various rotation speeds varying from 400 to 2800 rpm. Prior to electrochemical testing, the electrolyte was purged with an oxygen gas for at least 30 min and the O_2 flow was maintained above the electrolyte during the experiment to ensure an O_2-saturated solution. The electrochemical stability was performed via chronoamperometry test at a fixed potential of 0.1 V (*vs*. Hg/HgO) for 10,000 s.

The Koutecky–Levich (K-L) equation[2,3] was investigated to determine the electron transfer number (*n*) as follows:

$$\frac{1}{J} = \frac{1}{J_K} + \frac{1}{J_L} = \frac{1}{J_K} + \frac{1}{\beta\omega^{0.5}} \qquad (1)$$

WhereJ_L and J_K represent the limiting and kinetic currents, respectively. ω is the rotation speed of the working electrode, and β is the slope of Koutecky–Levich curve provided by:

$$\beta = 0.62nFC_oD_o{}^{2/3}\vartheta^{-1/6} \qquad (2)$$

D_o and C_o represent the oxygen diffusion coefficient (1.73*10^{-5} cm^2 s^{-1}) and oxygen concentration (1.21*10^{-3}mol cm^{-3}), respectively. $n$is the number of electron transfer, F is the Faradic constant (96,486 C mol^{-1}), and ϑ is the kinematic viscosity (0.01 cm^2 s^{-1}).

The features of the rotating ring disk electrode (RRDE) were investigated to estimate the hydrogen peroxide intermediates (H_2O_2%) based on the following equations[4,5]:

$$HO_2^-(\%) = 200 * \frac{\frac{I_R}{N}}{I_D + \frac{I_R}{N}} \qquad (3)$$

$$(n) = \frac{4*I_D}{I_D + \frac{I_R}{N}} \qquad (4)$$

I_R and I_D are the ring and desk currents, respectively, and N is the efficiency of the Pt-ring (0.35).

Characterization N-GO/Co_3O_4 NR and polyhedron catalysts

The morphologies of the N-GO/Co_3O_4 NR and polyhedron catalysts were investigated via field emission scanning electron microscopy (FE-SEM, JEOL model 6500). The scanning electron microscope was operated at 15 keV in order to record better SEM micrographs.

Raman spectroscopy (HR Micro Raman spectrometer, Horiba, JobinYvon) was conducted using an Ar ion laser at 633 nm. A CCD (charge coupled device) camera detection system and the LabSpec-3.01C software package were used for data acquisition and analysis, respectively. To ensure the accuracy and precision of the Raman spectra, 10 scans of 5 s from 300 cm^{-1} to 1,600 cm^{-1} were recorded.

The high angular annular dark-field (HAADF)–scanning/transmission electron microscope system (STEM) micrographs of the N-GO/Co_3O_4 NR and polyhedron catalysts were recorded using a JEM-ARM200F-G instrument supplied with aberration correctors at the illumination and imaging lens systems to observe TEM/STEM images at high resolution. The HAADF–STEM microscope was also equipped with a monochromated electron gun and supported by electron energy-loss spectroscopy at a high-energy resolution.

Wide-angle powder X-ray diffraction (XRD) patterns of the N-GO/Co_3O_4 NR and polyhedron catalysts were measured using an 18 kW diffractometer (Bruker D8 Advance) with monochromatic Cu Kα radiation. The sample measurement was repeated three times under rotation at various angles (15°, 30° and 45°). The diffraction data were analyzed using the DIFRAC plus Evaluation Package (EVA) software with the PDF-2 Release 2009 databases provided by Bruker AXS. The standard diffraction data were identified according to the databases of the International Centre for Diffraction Data (ICDD). For poor quality diffraction data, the TOPAS package program provided by Bruker AXS 2009 for profile and structure analysis was applied to integrate various types of X-ray diffraction analyses by supporting all profile fit methods currently employed in powder diffractometry.

X-ray photoelectron spectroscopy (XPS) analysis of (i) N-GO/Co_3O_4 NRs, N-GO/Co_3O_4 polyhedrons, and N-GO powder was conducted on a PHI Quantera SXM (ULVAC-PHI) instrument (Perkin–Elmer Co., USA) equipped with Al Kα as an X-ray source for excitation

(1.5 mm × 0.1 mm, 15 kV, 50 W) under a pressure of 4×10^{-8} Pa. A thin film of the sample was deposited on a Si slide before the start of analysis.

The textural surface parameters (surface area, pore volume, and the pore size distrbution) of the N-GO/Co_3O_4 NR and polyhedron catalystswere determined by N_2 adsorption–desorption isotherms which were measured using a BELSORP MIN-II analyzer (JP. BEL Co. Ltd) at 77 K. Prior to N_2 adsorption/desorption process, the samples were pre-treated at 200 °C for 8 h under vacuum until the pressure was equilibrated to 10^{-3} Torr. Specific surface area (S_{BET}) was calculated using multi-point adsorption data from linear segment of the N_2 adsorption isotherms using Brunauer–Emmett–Teller (BET) theory. The pore size distribution was determined from the analysis of desorption branch of isotherm using the nonlocal density functional theory (NLDFT).

Thermogravimetric and differential thermal analyses (TG and DTA, respectively) of surface area and the pore structure were measured using a simultaneous DTA-TG Apparatus TG-60 (Shimadzu, Japan).

Density functional theory (DFT) is performed in accordance with the DMol3 of BIOVIA Dassault systems.[7,8] The exchange-correlation energy function was represented by the Perdew–Burke–Ernzerhof (PBE) formalism.[9] The Kohn–Sham equation was expanded in a double numeric quality basis set (DNP) with polarization functions. To consider the relativistic effect, the DFT Semi-core Pseudo-potentialswere used for the treatment of the core electrons of the doped clusters.[10] The orbital cutoff range and Fermi smearing were selected as 5.0 Å and 0.001 Ha, respectively. The self-consistent-field (SCF) procedures were performed to obtain well-converged geometrical and electronic structures at a convergence criterion of 10^{-6}a.u. The energy, maximum force, and maximum displacement convergence were set to 10^{-6} Ha, 0.002 Ha/Å, and 0.005 Å, respectively.

DFT was also used to calculate the electrostatic potential (ESP) distribution. The electrostatic site potential is a measure of the Coulomb interaction per unit charge experienced by an ion at a given position in space. Modeling was performed to show a physical quantitative survey at each point on the isosurfaces using a feature of the surface-charging map. Typically, the isosurfaces of the electron densities were colored on the basis of ESP intensities (ESPI) using a lattice representation in which the charges are mapped on the cubic lattice in the so called contour

where the ESP is calculated. The slab model was constructed with nine atomic layers of each catalyst. To compare the active center within the structure, oxygen atoms at the surface and subsurface layers were involved in the stoichiometric mode. EP was investigated over the range of −0.06 eV to +0.6 eV as shown in the optimized model

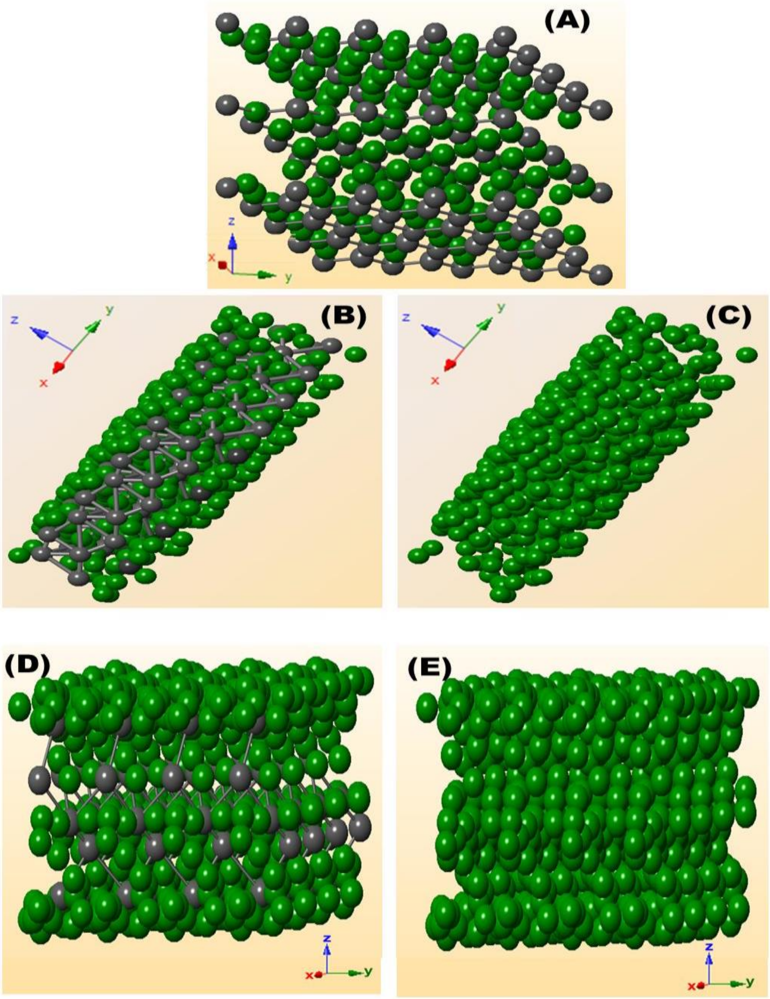

Figure S1 Schematic illustration of homogenously self-propelled particle-in-particle diffusion, interaction and growth protocol of N-GO/Co^{2+}-, Co^{3+}-ion and particle (surfactant-free) composite domains.The mechanistic formation of seedgrowth units of Co-particle(green color) into the GO mat surfaces under stirring-assisted diffusion (A-B-D) and heat-temperature treatment at 60 ^{o}C (C,E). (A) Diffusion of smaller Co^{2+} ions into the GO sheet after stirring (B, D), leading to theattachment of Co^{2+} ions onto both sides of graphene sheets. The continuous stirring under thermal treatment leads to form sandwich like-structures as growth units with diffusive–surface coverageCo $^{2+}$/GO aggregation.The top-view (A, D, E) or aside-view (B, C) atomic-atomic building structures onto GO sheet calculated by density functional theory (DFT).

Thermal stability of N-GO/Co_3O_4 polyhedron and NR single crystals

The thermal stabilities of the heterostructured N-GO/Co_3O_4 polyhedron and NR single crystals were performed using thermogravimetric analysis (TGA) under a steady flow of nitrogen gas at a heating ramp of 10 °C min^{-1} ranging from 25 to 600 °C (Figure S2). The observed weight loss at the region from 100 to 240 °C is attributed to the removal of water molecules and organic moieties. The sudden weight loss from 240 to 395 °C is equal to the loading of carbon component in the composite microstructure. Clearly, no weight loss was detected after 400 °C, indicating the successful formation of Co_3O_4 heteronanostructure at that temperature. The carbon degradation at a lower temperature is attributed to the diffusing/embedding into Co_3O_4 polyhedron and NR single crystals.[11,12]

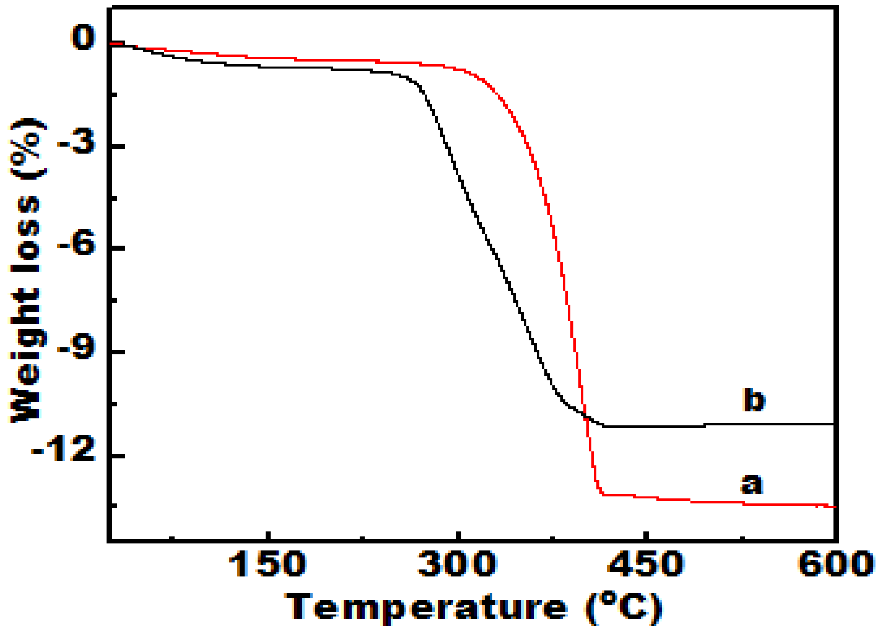

Figure S2 Thermogravimetric analyses (TGA) profiles of N-GO/Co_3O_4NR (a) and polyhedron (b) nanocrystals.

Mesoscopic features of N-GO/Co_3O_4 polyhedron and NR nanocrystals

The specific surface area and pore sizes of the mesoscopic N-GO/Co_3O_4 polyhedron and NR nanocrystals were determined by N_2-adsorption/desorption isotherms (Figure S3A−B). The samples show IV-type isotherm with hysteresis loop at relative pressures (P/P_0) in regions from 0.4 to 0.8 (Figure S3A). The pore size distribution curves (Figure S3B) of the samples show evidence of well -developed mesoporous structures with pore sizes of 21.46 and 17.27 nm for N-GO/Co_3O_4 polyhedrons and N-GO/Co_3O_4 nanorods, respectively. Furthermore, the Brunauer-Emmett-Teller (BET) surface area was found to be 44.14 and 56.5 m^2 g^{-1}. The mesoporous structures of the as-prepared materials originated from the self-assembly of the nanoparticles, which can effectively enhance the adsorption of oxygen molecules at the active site and is beneficial to the ORR process.

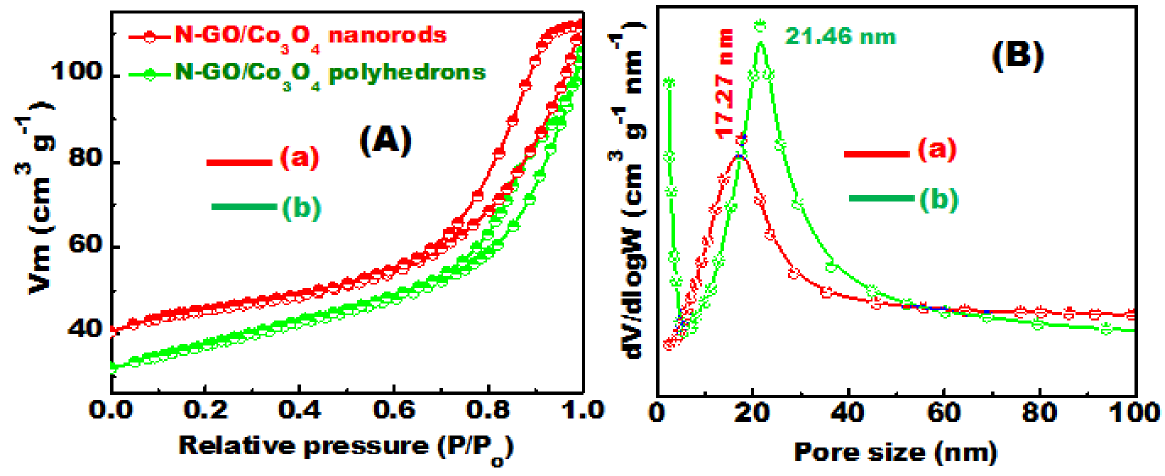

Figure S3 (A) N_2 adsorption/desorption isotherms recorded at 77 K, and (B) the related pore size distribution curves analyzed by NLDFT.

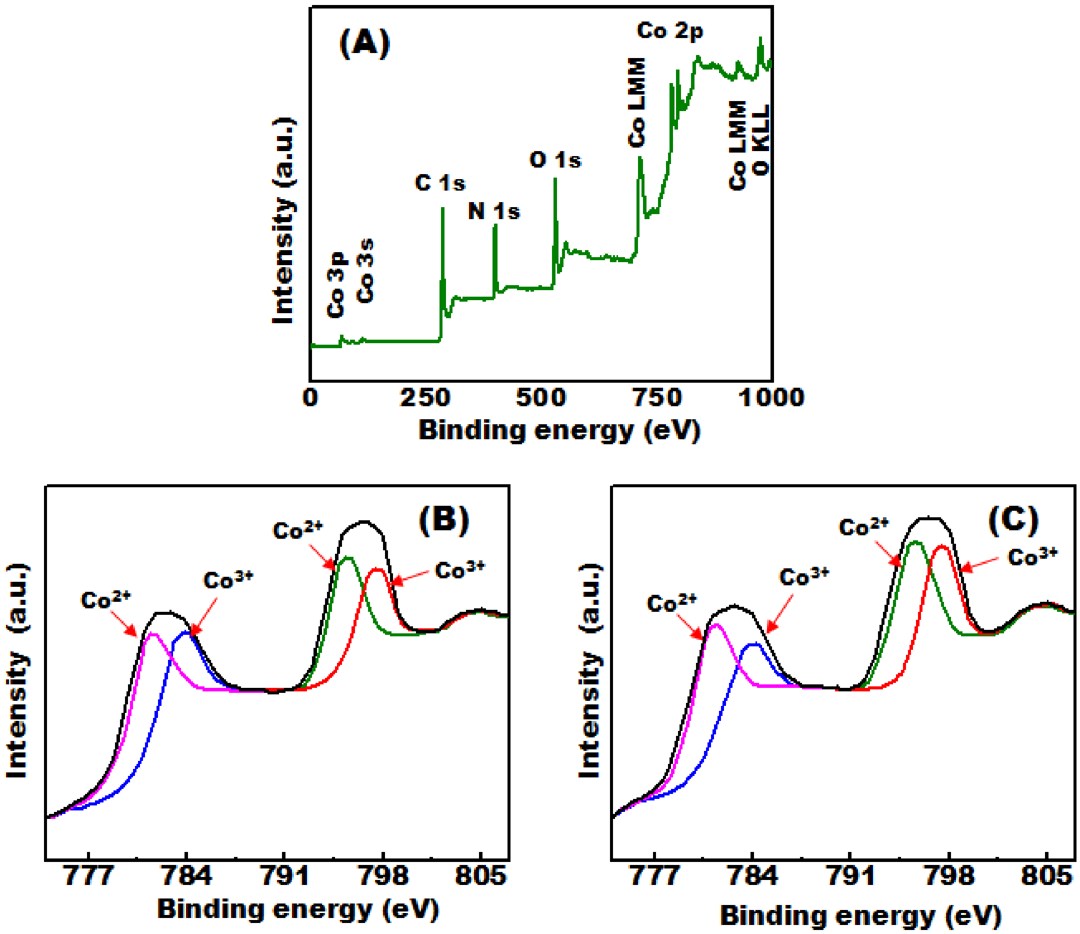

Figure S4 (A) Wide-XPS survey of N-GO/Co_3O_4 polyhedrons showing the existence of cobalt, oxygen, carbon, and nitrogen elements in the constructed hybrid. (B and C) The deconvolution of Co2p spectra for N-GO/Co_3O_4 polyhedrons (B) and N-GO/Co_3O_4 NRs (C)

Table S1 Summary of oxygen reduction reaction (ORR) parameters, including half wave potential ($E_{1/2}$) and diffusion-limiting current (J_L) values at 1600 rpm reported for nonprecious-transition-metal-based catalysts.

Catalyst	Catalyst loading (mg/cm^2)	$E_{1/2}$ (V vs. RHE)	J_L (mA cm^{-2})	Reference
N-CoO/graphene	unknown	~0.81	~1.8	13
$NiCo_2O_4$ NWs	unknown	~0.76	~6.0	14
Co_3O_4/rGO	0.17	~0.84	~5.10	15
Fe_3O_4/N-GAs	0.01	~0.68	~4.48	16
$NiCo_2O_4$	~0.8	~0.83	~1.6	17
Ni−Co_3O_4 NW array	0.08	~0.86	~5.76	18
$NiCo_2O_4$/graphene foam	0.4	~0.86	~6.2	19
$NiCo_2O_4$/rGO	0.4	~0.78	~1.6	20
$NiCo_2O_4$/graphene	0.4	~0.86	~6.2	21
N@carbon nanosheets	0.6	~0. 87	~5.8	22
N-GO/Co_3O_4 polyhedrons	~0.142 mg/cm^2	~ 0.81	~5.33	This study

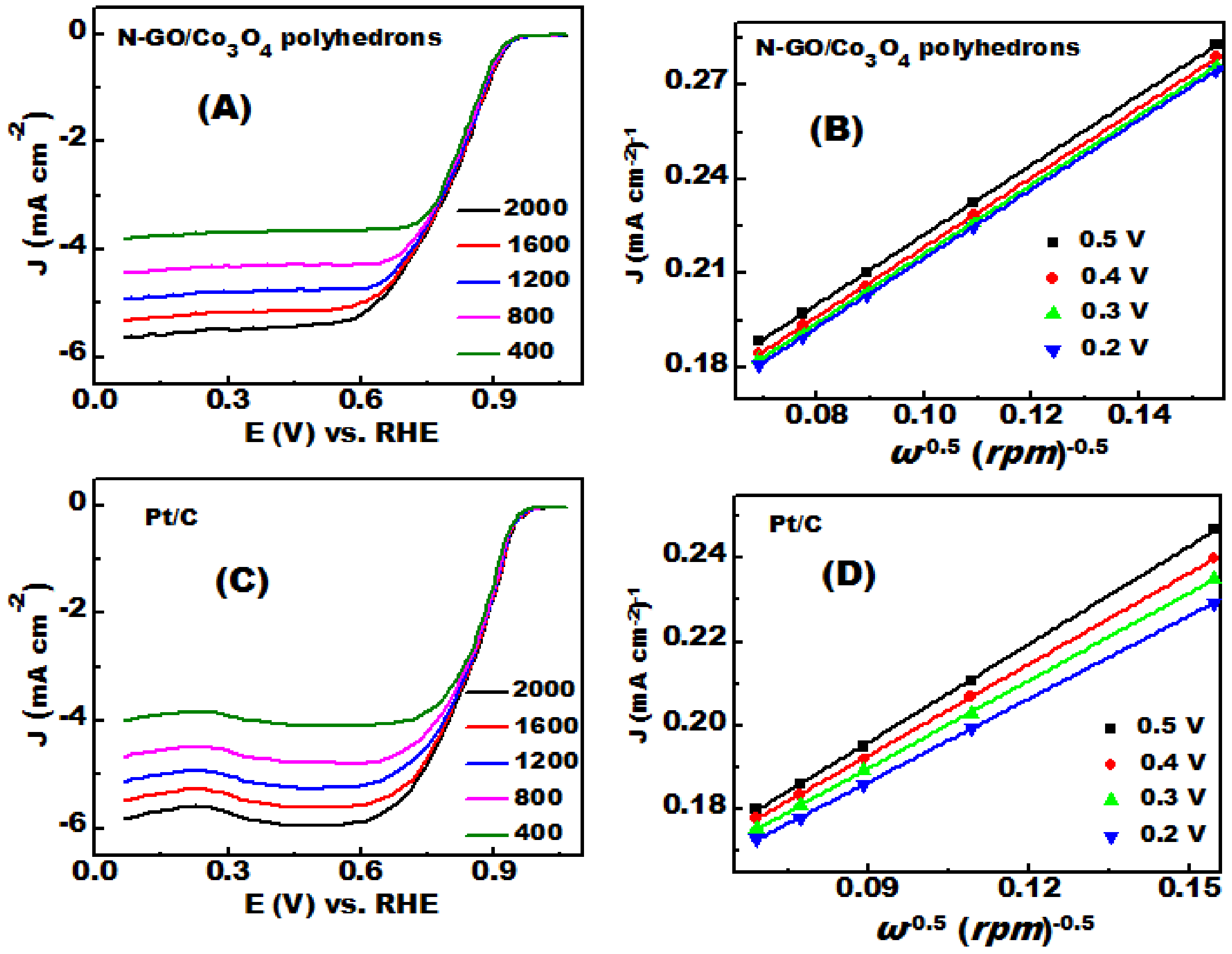

Figure S5 The LSV analysescollected in 0.1 M KOHO_2-saturatedsolutionat room temperatureusing rotating disk electrode at different rotating speeds and the corresponding K-L plots of N-GO/Co_3O_4 polyhedrons (A, B) and commercial Pt/C catalyst(C, D).

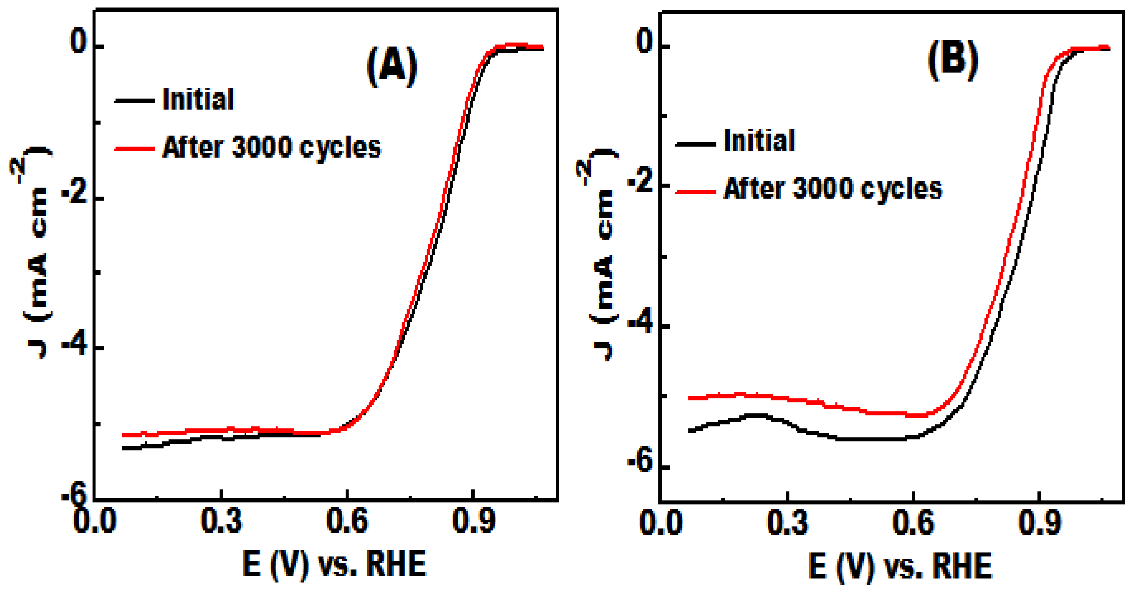

Figure S6 Durability test after potential cycling for 3000 cycles measured in O_2-saturated 0.1 M KOH solution for N-GO/Co_3O_4 polyhedron (A), and commercial Pt/C (B).

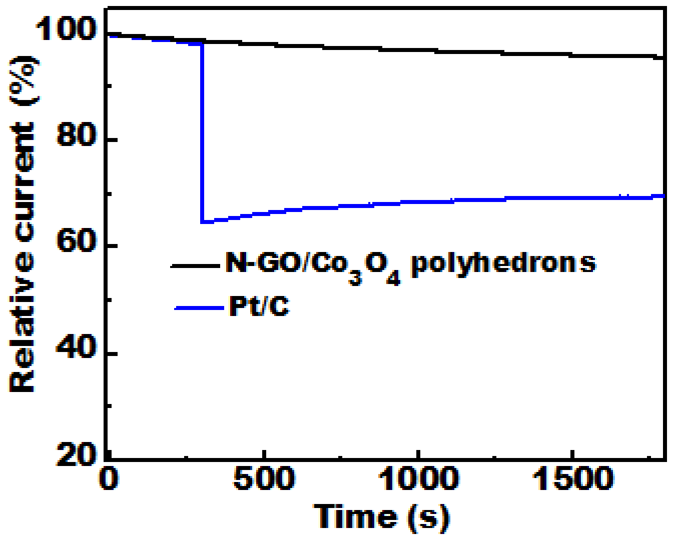

Figure S7 Methanol tolerance of N-GO/Co_3O_4 polyhedron and commercial Pt/C catalysts obtained by chronoamperometric responses upon insertion of methanol (3 M) for 2000 s at 1600 rpm.

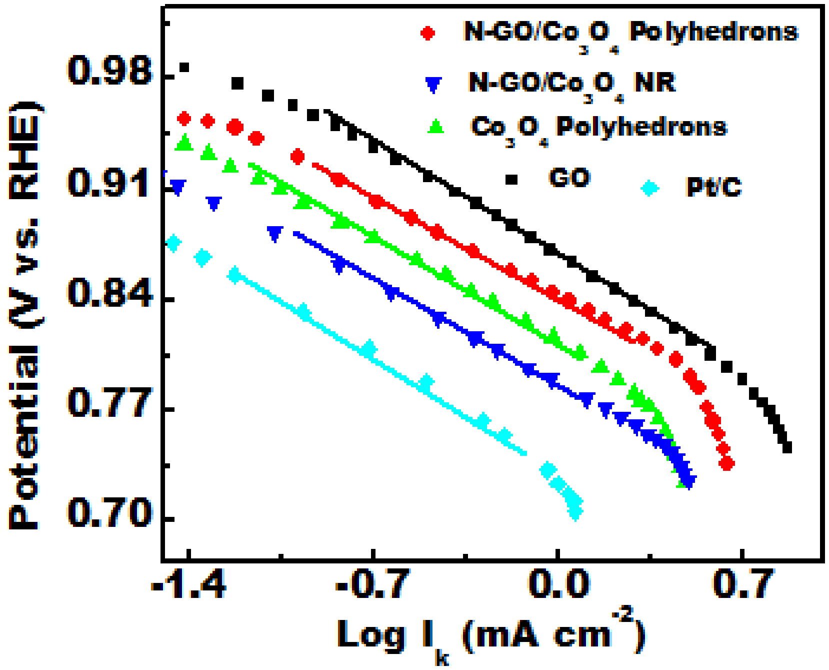

Figure S8 Analyzed Tafel plots of the examined electrocatalysts based on the kinetic current density (I_k) obtained from the Koutecky−Levich equation.

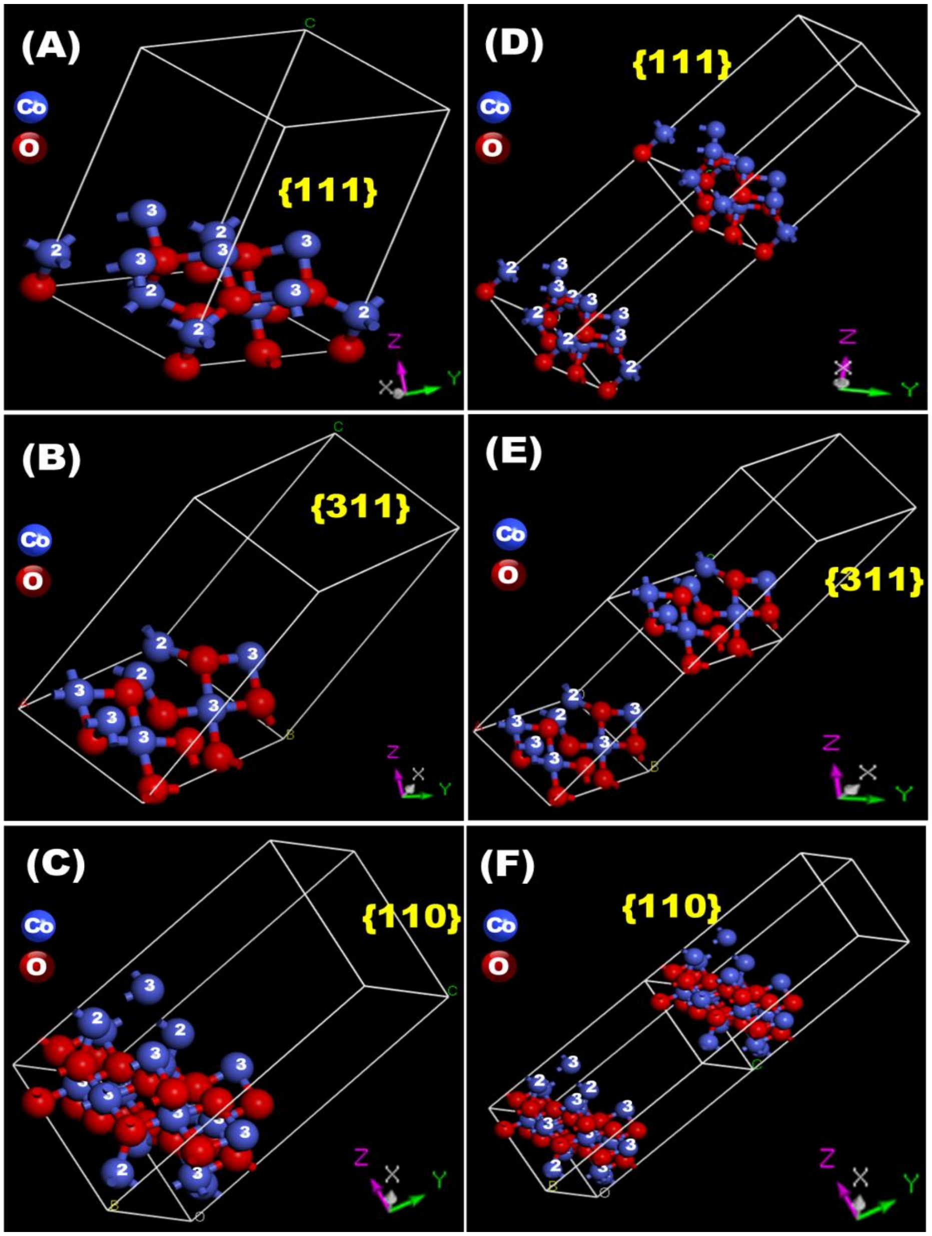

Figure S9 DFT calculations of the optimized atomic structure configurations oriented along {110}-NR, and along {111- and {113}-polyhedron crystal planes. The DFT show the location and change distribution of Co^{2+}, Co^{3+}, and O^{2-} atoms in the upper-top-surfaces of the {110}-NRs, and the {111}- and {113}-polyhedrons. The top-two-layers along each crystal orientation indicates the dense and stable configuration of Co^{2+}, Co^{3+}, and O^{2-} atoms in the whole surface and centre or crystal planes without distortion or defects during the formation of N-GO/Co_3O_4 catalysts.

References

1. Marcano, D.C. et al. Improved synthesis of graphene oxide. *ACS Nano* **4**, 4806−4814 (2010).
2. Cheon, J.Y.et al. Ordered mesoporous carbon–carbon nanotube nanocomposites as highly conductive and durable cathode catalyst supports for polymer electrolyte fuel cells. *J. Mater. Chem. A* **1**, 1270-1283 (2013).
3. Xiao, Y.P., et al. Self-deposition of Pt nanocrystals on Mn_3O_4 coated carbon nanotubes for enhanced oxygen reduction electrocatalysis. *J. Mater. Chem. A* **1**, 7463-7468(2013).
4. Choi, C.H., Park, S.H. & Woo, S.I. Phosphorus–nitrogen dual doped carbon as an effective catalyst for oxygen reduction reaction in acidic media: effects of the amount of P-doping on the physical and electrochemical properties of carbon. *J. Mater. Chem.* **22**, 12107-12115 (2012).
5. Hancock, C.A. Ong, A.L., Slater, P.R. & Varcoe, J.R. Development of $CaMn_{1-x}RuxO_{3-y}$ (x= 0 and 0.15) oxygen reduction catalysts for use in low temperature electrochemical devices containing alkaline electrolytes: ex situ testing using the rotating ring-disk electrode voltammetry method. *J. Mater. Chem. A* **2**, 3047- 3056 (2014).
6. Hassan, D.K., El-safty, S.A., Khalil, K.A., Dewidar, M. & Abu el-maged, G. Carbon supported engineering $NiCo_2O_4$ hybrid nanofibers with enhanced electrocatalytic activity for oxygen reduction reaction. *Materials* **9**, 759 (2016).
7. Delley, B. An all-electron numerical method for solving the local density functional for polyatomic molecules. *J. Chem. Phys*. **92**, 508–517 (1990).
8. Delley, B. Hardness conserving semilocal pseudopotentials. *Phys. Rev. B* **6**6, 155125 (pp.1-9) (2002).
9. Perdew, J. P.,Burke, K.& Ernzerhof, M. Generalized gradient approximation made simple. *Physical Review Letters* 77, 3865–3868 (1996).
10. Delley, B. From molecules to solids with the DMol3 approach. *J. Chem. Phys*. **113**, 7756–7764 (2000).
11. Wu, G. et al. Nitrogen-doped graphene-Rich catalysts derived from heteroatom polymers for oxygen reduction in non aqueous lithium O2 battery cathodes. *ACS Nano* **6**, 9764-9776 (2012).

12. Han, J., Kim, H., Kim, D.Y., Jo, S.M. & Jang, S. Water-soluble polyelectrolyte-grafted multiwalled carbon nanotube thin films for efficient counter electrode of dye-sensitized solar cells. *ACS Nano* **4**, 3503-3509 (2010).
13. Mao, S., Wen, Z., Huang, T., Hou, Y., Chen, J. High performance bi-functional electocatalysts of 3D crumpled graphene cobalt oxide nanohybrids for oxygen reduction and evolution reactions. *Energy Environ. Sci*. **7**, 609−616 (2014).
14. Jin, C., Lu, F., Cao, X.; Yang, Z., Yang, R. Facile synthesis and excellent electrochemical properties of $NiCo_2O_4$ spinel nanowire arrays as a bifunctional catalyst for the oxygen reduction and evolution reaction. *J. Mater. Chem. A* **1**, 12170−12177 (2013).
15. Liang, Y., Li, Y., Wang, H., Zhou, J., Wang, J., Regier, T., Dai, H. Co_3O_4 nanocrystals on graphene as a synergistic catalyst for oxygen reduction reaction. *Nat. Mater*. **10**, 780−786 (2011).
16. Wu, Z., Yang, S., Sun, Y., Parvez, K., Feng, X., Müllen, K. 3D nitrogen-doped graphene aerogel-supported Fe_3O_4 nanoparticles as efficient electrocatalysts for the oxygen reduction reaction. *J. Am. Chem. Soc*. **134**, 9082−9085 (2012).
17. Xiao, Y., Hu, C., Qu, L., Hu, C., Cao, M. Three-dimensional macroporous $NiCo_2O_4$ sheets as a non-noble catalyst for efficient oxygen reduction reactions. *Chem.-Eur. J.* **19**, 14271−14278 (2013).
18. Tong, X., Xia, X., Guo, C., Zhang, Y., Tu, J., Fan, H. J., Guo, X. Efficient oxygen reduction reaction using mesoporous Ni-doped Co_3O_4 nanowire array electrocatalysts. *J. Mater. Chem. A* **3**, 18372−18379 (2015).
19. Xili T., Shuai, C., Congxiu, G., Xinhui, X. & Xiang,-Y.G. Mesoporous $NiCo_2O_4$ nanoplates on three-dimensional graphene foam as an efficient electrocatalyst for the oxygen reduction reaction. *ACS Appl. Mater. Interfaces* **8**, 28274–28282 (2016).
20. Zhang, G., Xia, B. Y., Wang, X., Lou, X. W. Strongly coupled $NiCo_2O_4$-rGO hybrid nanosheets as a methanol-tolerant electrocatalysts for the oxygen reduction reaction. *Adv. Mater*. **26**, 2408−2412 (2014).
21. Neburchilov, V., Wang, H. J., Martin, J. J., Qu, W. A review on air cathodes for zinc−air fuel cells. *J. Power Sources* **195**, 1271−1291 (2010).

22. Wei, W., Liang, H., Parvez, K., Zhuang, X., Feng, X., Möllen, K. Nitrogen-doped carbon nanosheets with size-defined mesopores as highly efficient metal-free catalyst for the oxygen reduction reaction. *Angew. Chem., Int. Ed*. **53**, 1570−1574 (2014).
